# Spatiotemporal dynamics of EEG microstate networks over the first two years of life: A multi-cohort longitudinal study

**DOI:** 10.1162/IMAG.a.59

**Published:** 2025-06-27

**Authors:** Priyanka Ghosh, Kirsten A. Donald, Guilherme V. Polanczyk, Josh Paul Rodriguez, The Germina Project Team, The Khula Project Team, The LEAP 1kD EEG Working Group, Elizabeth Shephard, Laurel J. Gabard-Durnam

**Affiliations:** Center for Cognitive and Brain Health, Northeastern University, Boston, MA, United States; Division of Developmental Paediatrics, Department of Paediatrics and Child Health, Red Cross War Memorial Children’s Hospital, University of Cape Town, Cape Town, South Africa; The Neuroscience Institute, University of Cape Town, Cape Town, South Africa; Division of Child & Adolescent Psychiatry, Department & Institute of Psychiatry, Faculdade de Medicina FMUSP, Universidade de Sao Paulo, Sao Paulo, Brazil; Departamento de Psicologia da Aprendizagem, do Desenvolvimento e da Personalidade, Institute of Psychology, University of São Paulo, São Paulo, Brazil

**Keywords:** EEG, longitudinal infant microstates, spatiotemporal dynamics, largescale brain networks, geocultural differences, early life development

## Abstract

Microstates, brief instances of distinct spatial topographies measured with electroencephalography (EEG), offer a novel approach to studying whole-brain network dynamics at a sub-second scale. While emerging literature is leveraging microstate dynamics in adults and children to understand mature largescale network function, the developmental trajectories of these networks during their rapid construction in infancy remain poorly understood. Magnetic resonance approaches have revealed much about largescale networks in sleep, but very little is known about functional network dynamics in awake, behaving infants. Using longitudinal resting-state EEG from 854 infants across 2 diverse cohorts, we identified conserved emergence of various network configurations (classes A–G) during the first 2 years of life via data-driven clustering analyses. Significant longitudinal changes included more frequent and rapid transitions between microstate classes, particularly in early infancy. Sensory microstates showed consistent development across cohorts, while higher-order cognitive microstates demonstrated context-specific trends. These findings reveal novel insights into the functional development and organization of largescale brain networks during this period of substantial development.

## 1 Introduction

The first years of life represent a period of rapid and significant brain development, characterized by initial stages of functional maturation in the largescale brain networks (Fair et al., 2009;Gabard-Durnam & McLaughlin, 2020;Paterson et al., 2006;Uddin et al., 2023). These networks underlie interactions across widely distributed brain regions that support a range of sensory, cognitive, and socioemotional functions through coordinated neural activity (Bressler & Menon, 2010;Holz et al., 2023;Supekar et al., 2009). Recent approaches suggest the presence of dynamic interactions between largescale networks that unfold rapidly on a scale of milliseconds to seconds to support behavior (Allen et al., 2012;Khazaei et al., 2023;Laumann et al., 2024). However, most research on largescale brain network development during the first years of life relies on functional magnetic resonance imaging (fMRI), which is limited by slower estimates of network dynamics, capturing changes on the scale of seconds to minutes, typically during sleep or sedation (Custo et al., 2017;Graham et al., 2015;Grayson & Fair, 2017;Hutchison et al., 2013;Smyser et al., 2011;Zalesky et al., 2014;Zhang et al., 2019). Thus, currently there is an extremely limited understanding of the spatiotemporal dynamics of largescale networks in awake, behaving infants and toddlers during this important period of brain and behavioral development (Desowska et al., 2024;Wang et al., 2021;Yates et al., 2023).

The temporal precision of electroencephalography (EEG) in indexing neural activity and suitability for awake recording from birth facilitates addressing the functional organization of the dynamic brain in early life (Bagdasarov et al., 2024a,2024b;Brown & Gartstein, 2023;Hermans et al., 2024). Specifically, microstates, metastable EEG patterns lasting for brief intervals (60–120 ms), represent discrete brain states that capture instantaneous global brain network configurations and their dynamics (Hill et al., 2023;Khanna et al., 2015;Lehmann et al., 1987;Michel & Koenig, 2018;Tarailis et al., 2021). Microstates have distinct spatial (polarity-invariant) configurations representing largescale brain networks, and as this is a rapidly evolving field, there are some inconsistencies in the literature regarding their classification and functional interpretation (Kalburgi et al., 2024;Tarailis et al., 2023). However, they are typically labeled as class A (right anterior to left posterior, associated with the auditory network), B (left anterior to right posterior; visual network), C (frontal to occipital; Default Mode Network), and D (medial anterior to occipital; Dorsal Attention Network) (Britz et al., 2010;Koenig et al., 2002,^2024^;Michel & Koenig, 2018). Recent studies using data-driven approaches have identified additional microstates, including microstate maps E (centro-parietal maximum, associated with Salience Network), F (left-lateralized maximum; anterior DMN), and G (right-lateralized maximum; sensorimotor network) (Custo et al., 2017;Das et al., 2022;Tarailis et al., 2023). The temporal pattern of EEG microstates, that is, how frequently a given microstate is energetically dominant and the average duration of dominance, provides a window into the rapid, dynamic organization of brain networks in both health and disease (Hao et al., 2022;Khanna et al., 2014;Metzger et al., 2024;Nazare & Tomescu, 2024;Niu et al., 2024;Tomescu et al., 2015).

Preliminary cross-sectional studies in development indicate that EEG microstates may offer important insight into early development (Bagdasarov et al., 2022,2024b;Brown & Gartstein, 2023;Gui et al., 2021;Hill et al., 2023;Khazaei et al., 2021;Koenig et al., 2002). For example, microstates extracted from 6- to 10-month-old infants correlate with individual differences in temperament, suggesting early behavioral significance (Brown & Gartstein, 2023). Furthermore, infant microstate dynamics are also indicative of behavior measured years later as 8-month-old microstates measured during a social attention task predicted social skills and autism diagnoses at 3 years (Gui et al., 2021). These initial findings with cross-sectional microstate measurements establish their relevance for understanding largescale brain networks and emerging behavior (Adibpour et al., 2025;Bagdasarov et al., 2025;Gilbreath et al., 2025). However, the normative longitudinal development of microstate configurations and their dynamics over infancy remain unknown.

Our study aims to shed light on the development of largescale brain networks during infancy by analyzing the spatiotemporal dynamics of resting-state EEG microstates over the first 2 years of life. We conducted a multi-site longitudinal study using*a priori*harmonized EEG acquisitions across two cohorts (N = 854, observations = 2,314) in Cape Town, South Africa, and São Paulo, Brazil, which differ in geographic, cultural, demographic, and socioeconomic profiles. We then investigated the maturation of infant EEG microstates longitudinally as a function of age and sex in each cohort and compared the developmental patterns to assess their consistency across contexts. By tracking how the microstate dynamics change over infancy, this study seeks to improve our understanding of the neural underpinnings of early largescale brain network activity.

## 2 Methods

### 2.1 Participants

#### 2.1.1 Cohort Khula

In total, 394 mothers and their infants were recruited for this study (out of which 329 were enrolled antenatally) from Gugulethu, an informal settlement in Cape Town, South Africa, as part of a larger longitudinal project (Zieff et al., 2024). This sample is drawn from an impoverished community. Women were eligible to participate in the study if they were (i) in their third trimester of pregnancy (28–36 weeks) or up to 3 months postpartum, and (ii) over the age of 18 years at the time of recruitment. Inclusion criteria for the study included (a) singleton pregnancy, (b) no psychotropic drug endorsement during pregnancy, (c) no infant congenital malformation or abnormalities (e.g., spina bifida, Down’s syndrome), (d) no significant delivery complications (e.g., uterine rupture, birth asphyxia), and (e) gestational age 36 weeks or greater. Study demographics are provided inTable 1.*In Xhosa language, Khula means “grow,” signifying development.*

**Table 1.** Descriptive statistics of demographic data from the South Africa-based*Khula*study.

Cohort *Khula* (South Africa)	Visit 1 (2–6 months) (N = 242)	Visit 2 (5–12 months) (N = 249)	Visit 3 (12–18 months) (N = 261)	Visit 4 (19–26 months) (N = 218)	Overall (N = 318)
**Age (months)**					
Mean (SD)	3.74 (0.879)	8.72 (1.49)	14.1 (1.24)	21.5 (1.20)	5.27 (3.19%)
Median [Min, Max]	3.62 [1.97, 6.43]	8.57 [5.30, 12.2]	14.2 [11.6, 18.0]	21.4 [19.1, 25.7]	4.15 [1.97, 23.8]
**Child Sex**					
Male	125 (51.7%)	126 (50.6%)	135 (51.7%)	113 (51.8%)	162 (50.9%)
Female	117 (48.3%)	123 (49.4%)	126 (48.3%)	105 (48.2%)	156 (49.1%)
**Mother Education Level**					
Pre-primary	1 (0.4%)	0 (0%)	0 (0%)	0 (0%)	1 (0.3%)
Primary	12 (5.0%)	13 (5.2%)	12 (4.6%)	13 (6.0%)	14 (4.4%)
Secondary	199 (82.2%)	205 (82.3%)	217 (83.1%)	175 (80.3%)	262 (82.4%)
University/ College	30 (12.4%)	31 (12.4%)	32 (12.3%)	30 (13.8%)	41 (12.9%)
**Household Income**					
Less than R1000 per month	40 (16.5%)	47 (18.9%)	43 (16.5%)	38 (17.4%)	55 (17.3%)
R1000-R5000 per month	110 (45.5%)	115 (46.2%)	127 (48.7%)	108 (49.5%)	151 (47.5%)
R5000-R10000 per month	60 (24.8%)	55 (22.1%)	59 (22.6%)	46 (21.1%)	70 (22.0%)
More than R10000 per month	12 (5.0%)	9 (3.6%)	11 (4.2%)	7 (3.2%)	14 (4.4%)
Unknown	20 (8.3%)	23 (9.2%)	21 (8.0%)	19 (8.7%)	28 (8.8%)
**Ethnicity**					
South African	237 (97.9%)	247 (99.2%)	259 (99.2%)	217 (99.5%)	313 (98.4%)
African	5 (2.1%)	2 (0.8%)	2 (0.8%)	1 (0.5%)	5 (1.6%)
**Spoken Language**					
Xhosa	236 (97.5%)	243 (97.6%)	255 (97.7%)	214 (98.2%)	310 (97.5%)
Zulu	1 (0.4%)	1 (0.4%)	1 (0.4%)	0 (0%)	1 (0.3%)
Ndebele	1 (0.4%)	1 (0.4%)	1 (0.4%)	1 (0.5%)	1 (0.3%)
Afrikaans	0 (0%)	1 (0.4%)	1 (0.4%)	1 (0.5%)	1 (0.3%)
Sotho	2 (0.8%)	2 (0.8%)	2 (0.8%)	2 (0.9%)	2 (0.6%)
English	2 (0.8%)	1 (0.4%)	1 (0.4%)	0 (0%)	3 (0.9%)

Families were invited to participate in four in-laboratory study visits with EEG over their infant’s first 2 years of life. Not all infants contributed usable EEG at every visit. In total, 11.3% infants provided usable EEG data at only 1 time point, 13.5% infants provided 2 time points, 34% infants provided 3 time points, and 41.2% infants provided data at all 4 time points. The first visit occurred when infants were between approximately 2 and 6 months of age (mean age from usable data: 3.74 months; 51.7% males), the second visit when they were between 5 and 12 months (mean age: 8.72 months; 50.6% males), the third visit when they were between 12 and 18 months (mean age: 14.1 months; 51.7% males), and finally, the fourth visit when they were between 19 and 26 months of age (mean age: 21.5 months; 51.8% males). In total, 318 of the 394 enrolled infants contributed EEG data to the present study.

All procedures were approved by the Human Research Ethics Committee at the University of Cape Town in South Africa. Written consent was obtained from caregivers annually, giving caregivers more agency to opt out if they chose to discontinue participation at any point. Families were compensated with ZAR 300 (~USD 15) for their time after each visit. Additionally, transportation was made available to and from research sites for participants’ convenience. Food and beverages were also provided during each visit.

#### 2.1.2 Cohort Germina

In total, 557 mother–infant dyads were enrolled into a longitudinal cohort study in São Paulo, Brazil (for more information, see Fatori et al., 2024). This cohort is a middle–high socioeconomic status sample. Infants included in this analysis were recruited postnatally with the average age at recruitment being 2.4 months. Inclusion criteria were (a) maternal age of 20–45 years, (b) infant age of 3 months 0 days to 3 months and 29 days for the first visit, (c) gestational age of >37 weeks, (d) infant birth weight >2,000 grams, (e) no substance endorsement during pregnancy, (f) no history of severe maternal mental disorders (e.g., psychosis, bipolar disorder), (g) no delivery complications requiring medical intervention (e.g., perinatal asphyxia, shoulder dystocia, excessive bleeding), (h) no infant genetic syndrome or auditory/visual impairment diagnoses, and (i) availability for in-person laboratory visits. Study demographics are provided inTable 2.*In Portuguese, Germina means “to germinate,” signifying early growth.*

**Table 2.** Descriptive statistics of demographic data from the Brazil-based*Germina*study.

Cohort *Germina* (Brazil)	Visit 1 (3–4 months) (N = 349)	Visit 2 (5–10 months) (N = 444)	Visit 3 (10–17 months) (N = 350)	Visit 4 (18–30 months) (N = 201)	Overall (N = 536)
**Age (months)**					
Mean (SD)	3.62 (0.213)	7.64 (1.25)	13.2 (2.06)	21.1 (3.11)	5.41 (3.27)
Median [Min, Max]	3.61 [3.06, 4.01]	7.41 [5.06, 9.99]	12.9 [10.0, 17.0]	19.7 [18.0, 30.4]	3.81 [3.06, 28.8]
**Child Sex**					
Male	158 (45.3%)	217 (48.9%)	171 (48.9%)	101 (50.2%)	261 (48.7%)
Female	191 (54.7%)	227 (51.1%)	179 (51.1%)	100 (49.8%)	275 (51.3%)
**Mother Education Level**					
Primary	5 (1.4%)	4 (0.9%)	3 (0.9%)	2 (1.0%)	5 (0.9%)
Secondary	71 (20.3%)	96 (21.6%)	76 (21.7%)	40 (19.9%)	108 (20.1%)
University/College	271 (78.2%)	344 (77.5%)	271 (77.4%)	159 (79.1%)	423 (78.9%)
**Father Education Level**					
Primary	17 (4.9%)	17 (3.8%)	14 (4.0%)	6 (3.0%)	23 (4.3%)
Secondary	87 (24.9%)	112 (25.2%)	87 (24.9%)	61 (30.3%)	131 (24.4%)
University/ College	241 (69.1%)	310 (69.8%)	246 (70.3%)	131 (65.2%)	377 (70.3%)
Unknown	4 (1.1%)	5 (1.1%)	3 (0.9%)	3 (1.5%)	5 (0.9%)
**Household Income**					
Less than BRL1000 per month	19 (5.4%)	10 (2.3%)	3 (0.9%)	1 (0.5%)	20 (3.7%)
BRL1000-BRL5000 per month	131 (37.5%)	129 (29.1%)	97 (27.7%)	69 (34.3%)	182 (34.0%)
BRL5000-BRL10000 per month	80 (22.9%)	118 (26.6%)	107 (30.6%)	51 (25.4%)	136 (25.4%)
More than BRL10000 per month	118 (33.8%)	160 (36.0%)	126 (36.0%)	66 (32.8%)	183 (34.1%)
Unknown	1 (0.3%)	27 (6.1%)	17 (4.9%)	14 (7.0%)	15 (2.8%)
**Ethnicity**					
White	225 (64.5%)	286 (64.4%)	224 (64.0%)	129 (64.2%)	349 (65.1%)
Black	34 (9.7%)	40 (9.0%)	28 (8.0%)	18 (9.0%)	46 (8.6%)
Pardo	72 (20.6%)	92 (20.7%)	74 (21.1%)	42 (20.9%)	111 (20.7%)
Other	18 (5.2%)	26 (5.9%)	24 (6.9%)	12 (6.0%)	30 (5.6%)
**Spoken Language**					
Portuguese	344 (98.6%)	439 (98.9%)	347 (99.1%)	199 (99.0%)	529 (98.7%)
Spanish	2 (0.6%)	2 (0.5%)	1 (0.3%)	0 (0%)	2 (0.4%)
Bilingual	1 (0.3%)	0 (0%)	0 (0%)	0 (0%)	1 (0.2%)
English	0 (0%)	1 (0.2%)	1 (0.3%)	1 (0.5%)	2 (0.4%)
Unknown	2 (0.6%)	2 (0.5%)	1 (0.3%)	1 (0.5%)	2 (0.4%)

Families were invited to participate in four in-laboratory study visits with EEG over their infant’s first 2 years of life. Not all infants contributed usable EEG at every visit. In total, 15.1% infants provided usable EEG data at only 1 time point, 33% infants provided 2 time points, 37.9% infants provided 3 time points, and 14% infants provided data at all 4 time points. The first visit occurred when infants were between approximately 3 and 4 months of age (mean age from usable data: 3.62 months; 45.3% males), the second visit when they were between 5 and 10 months (mean age: 7.64 months; 48.9% males), the third visit when they were between 10 and 17 months of age (mean age: 13.2 months; 48.9% males), and the fourth visit when they were between 18 and 30 months of age (mean age: 19.7 months; 50.2% males). EEG data from 536 of the 557 enrolled infants were deemed usable for the study.

All procedures were approved by the Ethics Committee for the Analysis of Research Projects (CAPPESq) and the National Council of Ethics in Research (ref.: CAAE 49671221.2.0000.0068). In accordance with the Declaration of Helsinki, all mothers provided written informed consent before completing any study measure.

### 2.2 EEG data acquisition

Baseline EEG was recorded at each visit using largely harmonized a priori protocols across visit ages and between sites. Infants were seated on their caregiver’s lap approximately 60 cm in front of a computer monitor (30 x 45.5 cm, 1440 x 900-pixel resolution) throughout recording where they passively viewed a silent screensaver-like video of moving shapes as inFox et al. (2024). The video was programmed and presented in E-Prime (Psychology Software Tools, Inc., Sharpsburg, PA). Video coding of looking to screen was not available during the 2–3 minute paradigm in these cohorts. Recordings took place in a dimly lit, quiet room without electrical shielding. Two-minute (*Germina*) and 3-minute (*Khula*) long EEG were recorded using a 128-channel HydroCel geodesic sensor net and a Net Amps 400 amplifier (Magstim EGI, Whitland, UK). The caregivers were instructed to sit silently during the session. Data were referenced online to the vertex electrode Cz and sampled at 500 Hz (*Germina*) and 1,000 Hz (*Khula*) via NetStation software (Magstim EGI, Whitland, UK). Electrode impedances were kept below 100 kΩ wherever possible in accordance with the capabilities of the high-impedance amplifiers. In*Khula*, nets designed with modified taller pedestals were used as needed for improving the inclusion and experience of infants with Afro-textured hair (Mlandu et al., 2024). Shea moisture leave-in castor oil conditioner was applied to hair across the scalp prior to net placement to improve both impedances and participant comfort. The leave-in conditioner is insulating to prevent electrical bridging, has not been found to disrupt the EEG signal during testing, and allows for nets to lay closer to the scalp for Afro-textured hair types, while making it far more comfortable to remove from the scalp at the end of testing (Fox et al., 2024;Mlandu et al., 2024).

### 2.3 EEG data preprocessing

EEG data were converted from native Netstation .mff format to .raw format to remove identifying video information across sites. The .raw files were processed using the Harvard Automated Processing Pipeline for EEG (HAPPE), an automated open-source EEG preprocessing software customized for the developmental population (Gabard-Durnam et al., 2018;Monachino et al., 2022). HAPPE v 4.1 was run using MATLAB (2022b) and EEGLAB (Delorme & Makeig, 2004) with the preprocessing parameters as listed inSupplementary Table S1. As commonly used for microstate analysis, the data were filtered between 1 and 40 Hz (Britz et al., 2010) with a finite impulse response (FIR) bandpass filter in HAPPE. The outer rim electrodes of the net were removed from analysis, which is a common practice in infant EEG research given their susceptibility to artifact contamination (seeMonachino et al., 2022). Bad channel detection was performed on remaining channels. Electrical line noise was removed at 50 Hz from the*Khula*data and at 60 Hz from the*Germina*data using CleanLine (Mullen, 2013) via a multi-taper regression which can remove electrical noise without distortion of the EEG signal in the nearby frequencies, unlike traditional notch filters (Mitra & Pesaran, 1999). Artifacts were corrected with primarily wavelet thresholding, but also MuscIL (independent component analysis restricted to muscle-classified artifacts) within HAPPE. Bad channels were interpolated followed by rereferencing of the data to the average reference. The continuous EEG recording from each participant was epoched into 2-second-long segments and any segment with amplitude change ±150 µV was further rejected. SeeSupplementary Table S2for EEG data quality measures across sites.

### 2.4 Microstate computations

Resting-state EEG microstates in infants were computed from the preprocessed EEG data using the*generateMicroStates.m*script of HAPPE v4.1, which is based on functions from EEGLAB (Delorme & Makeig, 2004) and the microstate toolbox plugin (MST version 1.0) (Poulsen et al., 2018). Participants who retained more than 15 segments (i.e., 30 seconds of data) after preprocessing were considered for further analysis. Given the paucity of literature on pre-processing for microstates in early development (though seeBagdasarov et al., 2024a), additional sensitivity analyses were conducted to test whether the identity and number of microstate classes identified in the data varied as a function of retained segments by re-computing microstates using only infants with 100% retained segments (91 segments in at least 30 infants). These restricted analyses returned the same number and type of microstate classes as reported with the full sample, suggesting microstate determination was robust to segment retention variance within this developmental sample and consistent with prior research on the matter (Bagdasarov et al., 2024a).

For each cohort, microstate analysis was performed for each visit (time point) separately to determine whether identity and number of microstate classes varied over early development. To identify microstate classes, all available segments from a participant were concatenated and treated as one continuous signal. Datapoints corresponding to GFP (Global Field Power) maxima, which represent maps with a high signal-to-noise ratio, were subjected to a modified k-means clustering algorithm (Koenig et al., 2002;Pascual-Marqui et al., 1995) to identify microstate prototypes (within the range of 2 to 8 prototypes) in a polarity invariant manner. This means that the algorithm did not differentiate between spatially proportional but oppositely polarized topographical maps when assigning microstate clusters (Pascual-Marqui et al., 1995). The assumption in clustering is that all EEG data assigned to the same cluster originate from neural generators underlying the prototype topography of that cluster. The clustering process was initialized 50 times stochastically, with a maximum of 1,000 iterations per run. A total of 1,000 GFP peaks per participant were included in the segmentation, with a minimum peak distance of 10 ms, as recommended byPoulsen et al. (2018). Visual inspection of the topographies and evaluation of fit measures, including global explained variance (GEV) and the cross-validation (CV) criterion (Poulsen et al., 2018), revealed five to six prototypical microstate maps (classes A to G) that provided an optimal clustering solution depending on the ages being clustered. While higher GEV indicated better explained variance, a lower CV criterion reflected less residual noise and was thus desirable (for measure of fit plots, seeSupplementary Materials S5–S12). This data-driven selection represented a qualitative balance between specificity and generalizability across cohorts and time points, where the former typically improved by increasing the number of microstates and the latter by limiting their number (Hill et al., 2023;Michel & Koenig, 2018).

The microstate classes obtained were subsequently backfitted to each participant’s EEG data. To mitigate the influence of noise inherent in spontaneous EEG recordings, which can result in consecutive short time frames being labeled as different classes (Musaeus et al., 2019;Poulsen et al., 2018), we applied the default temporal smoothing approach in the microstate toolbox and rejected microstate segments shorter than 30 ms (Poulsen et al., 2018). This method reclassifies the labels of those segments to the next best-fitting microstate, as measured by global map dissimilarity (Poulsen et al., 2018). The following statistical parameters were then extracted from the microstate labeled data corresponding to the various microstate classes at each visit: (a)*Duration,*defined as the average duration in milliseconds for which a given microstate class remains stable; (b)*Occurrence*determines the average number of times a microstate class is dominant within 1 second; (c)*Coverage*reflects the fraction of time for which a given microstate is active uninterruptedly; and (d)*GEV*or the global explained variance is the variance in the EEG data explained by a particular microstate class.

### 2.5 Statistical analyses

The mean and the standard deviation of each microstate parameter (i.e., occurrence, duration, coverage, GEV) were computed across participants within each cohort for each of the microstate classes for all four visits. Datapoints lying outside 3 standard deviations from the mean for a given microstate feature for each visit within cohort were considered outliers and were not included in the analyses. Using this criterion, 11 data points were removed from*Khula*analyses (1 at*time point 1*, 3 at*time point 2*, none at*time point 3*, and 7 at*time point 4*) and 6 data points were removed from*Germina*analyses (none at*time point 1*, 1 at*time point 2*, 2 at*time point 3*, and 3 at*time point 4*).

Primary statistical analyses focused on duration and occurrence features for the microstate classes present across all four visits in each cohort. For each cohort, the developmental changes in the durations and the occurrences of the microstate classes were analyzed with linear mixed effect models using the*lme4*package in*R version 2024.04.2+764*(R Core Team, 2024). The model parameters were estimated using maximum likelihood (Bates et al., 2015). Random subject intercepts were included in all models to account for between-participant variability in initial microstate characteristics. A fixed effect of sex was also modeled, and the number of retained segments at each visit was included as a covariate of no interest given prior research suggesting some microstate features’ internal consistency varies with segment number (Bagdasarov et al., 2024a). Retained segment number had a negligible effect size in all models (*f^2^*< 0.01).

To assess the best-fitting model for the age-associated growth trajectories of microstate features within each cohort, three models of age-related changes were run for each feature that included (a) linear age term, (b) linear and quadratic age term, (c) linear and logarithmic age term. The fits of these three models were compared (*linear*vs.*linear+quadratic*vs.*linear+log*) using BIC (Bayesian Information Criterion) to reduce the risk of over-fitting data by penalizing the more complicated models for additional terms. A non-linear, more complex model was selected if there was strong support for inclusion of the non-linear term (i.e., BIC difference of six points or greater), and if both nonlinear term models (*linear+quadratic*vs.*linear+log*) met the BIC criterion for support, the model with the lowest BIC was chosen as the best fit to the data (Raftery, 1995).

## 3 Results

### 3.1 Developmental changes in microstate configurations

As shown inFigure 1, polarity-invariant canonical microstates A, B, C, D, and E were seen in individuals at 2–6 months (N = 242) as well as at 5–12 months (N = 249) in the Cape Town (*Khula*) cohort. Microstate C was replaced with the emergence of two additional microstates F and G at 12–18 months (N = 261) and 19–26 months (N = 218) in the*Khula*cohort. In the Brazil (*Germina*) cohort, microstates A, B, C, D, and E were seen at 3–4 months (N = 349), 5–10 months (N = 444), and 10–17 months (N = 350). Microstate C was replaced with the emergence of microstates F and G at 18–30 months (N = 201). Microstate statistics for all classes (A through G) at each time point for each cohort are depicted inTable 3.

**Fig. 1.**
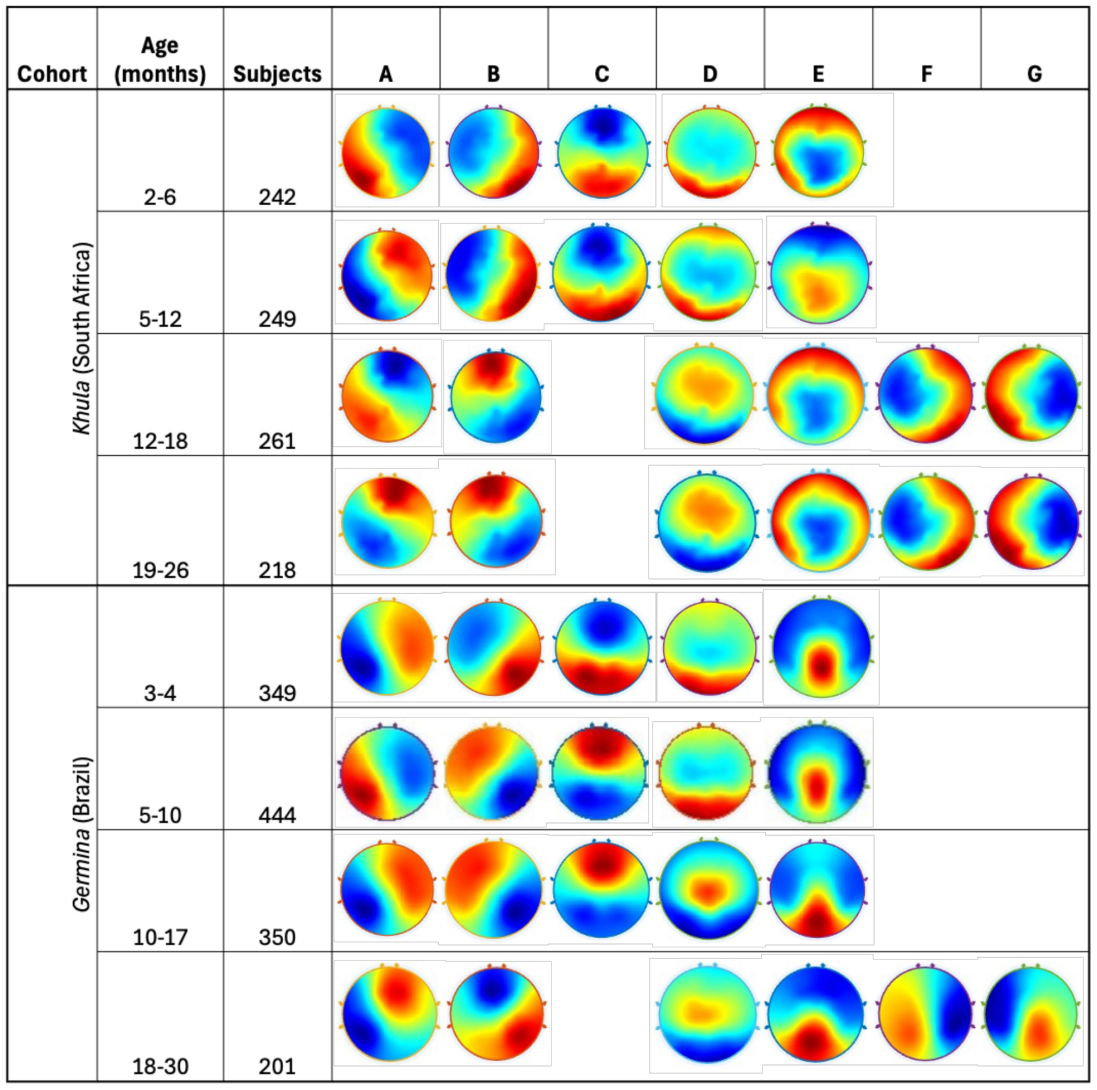
Infant microstates. The figure represents open-eyes resting-state EEG microstate topographies (polarity-invariant) of infants recorded at four different developmental time windows longitudinally across the first 2 years of life from South Africa-based*Khula*and Brazil-based*Germina*cohorts.

**Table 3.** Summary statistics of microstate parameters.

Cohort	*Khula* (South Africa)				*Germina* (Brazil)			
Time point	Visit 1 (2–6 months)	Visit 2 (5–12 months)	Visit 3 (12–18 months)	Visit 4 (19–26 months)	Visit 1 (3–4 months)	Visit 2 (5–10 months)	Visit 3 (10–17 months)	Visit 4 (18–30 months)
**occurrenceA (/s)**	2.38 (0.39); 1.59–3.49	2.59 (0.41); 1.51–3.77	2.57 (0.42); 1.48–3.73	2.52 (0.39); 1.32–3.55	2.31 (0.32); 1.47–3.2	2.25 (0.32); 1.52–3.13	2.45 (0.35); 1.61–3.41	2.35 (0.42); 1.13–3.63
**occurrenceB (/s)**	2.29 (0.34); 1.4–3.33	2.38 (0.34); 1.54–3.35	2.63 (0.40); 1.42–3.83	2.61 (0.40); 1.44–3.75	2.39 (0.39); 1.35–3.42	2.36 (0.32); 1.49–3.28	2.49 (0.37); 1.51–3.53	2.4 (0.49); 1.20–3.84
**occurrenceC (/s)**	2.55 (0.39); 1.52–3.68	2.97 (0.34); 2.12–3.86	–	–	2.52 (0.35); 1.56–3.43	2.67 (0.35); 1.72–3.71	2.83 (0.39); 1.76–3.84	–
**occurrenceD (/s)**	2.3 (0.35); 1.45–3.3	2.16 (0.38); 0.98–3.04	2.45 (0.39); 1.19–3.35	2.83 (0.33); 1.78–3.71	2.09 (0.32); 1.16–2.8	2.44 (0.33); 1.35–3.21	2.18 (0.56); 0.47–3.41	2.02 (0.54); 0.45–3.11
**occurrenceE (/s)**	2.06 (0.31); 1.17–2.93	2.23 (0.38); 1.2–3.3	1.79 (0.42); 0.55–2.77	1.73 (0.39); 0.71–2.86	2.09 (0.33); 1.11–2.94	2.09 (0.32); 1.13–3.08	2.41 (0.29); 1.61–3.26	2.5 (0.42); 1.46–3.59
**occurrenceF (/s)**	–	–	1.91 (0.27); 1.29–2.59	2.01 (0.29); 1.22–2.83	–	–	–	2.07 (0.32); 1.16–2.88
**occurrenceG (/s)**	–	–	1.91 (0.24); 1.27–2.61	2.02 (0.29); 1.18–2.68	–	–	–	2.01 (0.34); 1.07–2.87
**durationA (ms)**	82.91 (8.43); 64.98–109.99	79.67 (7.82); 63.57–102.28	78.93 (8.24); 57.06–105.25	73.66 (6.06); 60.1–90.73	82.31 (7.19); 70.2–107.06	78.21 (7.06); 64.11–101.3	76.42 (7.01); 60.32–99.47	74.41 (7.28); 59.09–95.28
**durationB (ms)**	80.97 (7.65); 62.17–106.04	74.47 (5.78); 63.38–92.76	80.14 (8.25); 56.24–105.38	74.87 (6.82); 59.67–95.54	83.58 (7.02); 62.43–107.33	79.69 (6.53); 66.97–101.42	76.56 (6.84); 61.32–99.57	75.11 (6.35); 57.14–95.15
**durationC (ms)**	93.26 (8.74); 70.94–118.83	92.37 (9.36); 70.28–119.67	–	–	93.94 (8.44); 70.59–118.15	91.37 (8.59); 69.56–117.04	86.54 (7.27); 69.04–109.32	–
**durationD (ms)**	87.9 (12.42); 62.26–127.2	76.26 (8.82); 55.15–98.83	78.51 (8.47); 56.63–101.94	81.13 (8.67); 63.56–106.59	86.93 (12.21); 61.58–125.31	87.44 (9.91); 59.12–115.01	80.1 (10.75); 52.34–112.63	72.82 (8.58); 54.09–102.33
**durationE (ms)**	81.76 (9.59); 64.07–112.09	76.16 (6.58); 59.09–95.03	68.97 (6.29); 53.52–90.2	65 (5.12); 52.62–82.14	87.05 (11.4); 62.18–125.33	81.94 (9.74); 58.5–116.24	80.62 (9.4); 62.14–113.09	79.29 (8.8); 60.71–115.38
**durationF (ms)**	–	–	68.22 (5.05); 55.11–85.29	66.52 (4.62); 57.82–81.79	–	–	–	69.16 (5.92); 58.18–89.1
**durationG (ms)**	–	–	68.45 (5.26); 58.48–85.4	66.87 (4.57); 58–80.51	–	–	–	69.93 (6.71); 56.33–91.71
**coverageA (%)**	0.2 (0.04); 0.11–0.3	0.21 (0.04); 0.11–0.34	0.2 (0.04); 0.11–0.35	0.19 (0.04); 0.08–0.3	0.19 (0.03); 0.11–0.29	0.18 (0.03); 0.08–0.27	0.19 (0.04); 0.09–0.29	0.17 (0.04); 0.06–0.31
**coverageB (%)**	0.19 (0.04); 0.1–0.29	0.18 (0.03); 0.09–0.28	0.21 (0.04); 0.08–0.35	0.2 (0.04); 0.1–0.31	0.2 (0.04); 0.11–0.31	0.19 (0.03); 0.11–0.29	0.19 (0.04); 0.11–0.3	0.18 (0.05); 0.07–0.31
**coverageC (%)**	0.24 (0.05); 0.12–0.35	0.27 (0.05); 0.17–0.41	–	–	0.24 (0.04); 0.12–0.35	0.24 (0.04); 0.14–0.36	0.25 (0.04); 0.15–0.37	–
**coverageD (%)**	0.2 (0.05); 0.07–0.36	0.17 (0.04); 0.04–0.29	0.19 (0.04); 0.05–0.31	0.23 (0.04); 0.1–0.36	0.18 (0.05); 0.06–0.33	0.21 (0.04); 0.08–0.34	0.18 (0.06); 0.02–0.33	0.15 (0.05); 0.03–0.3
**coverageE (%)**	0.17 (0.04); 0.07–0.27	0.17 (0.04); 0.07–0.28	0.12 (0.03); 0.03–0.22	0.11 (0.03); 0.04–0.22	0.18 (0.04); 0.08–0.31	0.17 (0.04); 0.07–0.3	0.19 (0.04); 0.08–0.31	0.2 (0.05); 0.1–0.38
**coverageF (%)**	–	–	0.13 (0.02); 0.08–0.19	0.13 (0.02); 0.07–0.19	–	–	–	0.14 (0.03); 0.09–0.23
**coverageG (%)**	–	–	0.13 (0.02); 0.09–0.19	0.14 (0.02); 0.08–0.2	–	–	–	0.14 (0.03); 0.06–0.24
**GEV A**	0.06 (0.02); 0.02–0.14	0.08 (0.03); 0.03–0.18	0.1 (0.04); 0.03–0.22	0.09 (0.03); 0.02–0.18	0.05 (0.02); 0.02–0.1	0.05 (0.01); 0.02–0.09	0.05 (0.02); 0.02–0.11	0.06 (0.02); 0.01–0.14
**GEV B**	0.05 (0.02); 0.02–0.11	0.05 (0.02); 0.02–0.1	0.11 (0.04); 0.02–0.23	0.1 (0.03); 0.03–0.18	0.06 (0.02); 0.02–0.12	0.06 (0.02); 0.02–0.11	0.06 (0.02); 0.02–0.12	0.07 (0.03); 0.02–0.16
**GEV C**	0.1 (0.04); 0.02–0.22	0.15 (0.05); 0.06–0.3	–	–	0.09 (0.04); 0.02–0.21	0.1 (0.03); 0.03–0.21	0.11 (0.04); 0.03–0.22	–
**GEV D**	0.07 (0.03); 0.01–0.17	0.05 (0.02); 0.01–0.1	0.09 (0.03); 0.02–0.18	0.13 (0.04); 0.04–0.24	0.04 (0.02); 0.01–0.1	0.06 (0.02); 0.01–0.13	0.04 (0.02); 0–0.1	0.04 (0.02); 0–0.09
**GEV E**	0.04 (0.01); 0.01–0.07	0.05 (0.02); 0.01–0.11	0.03 (0.01); 0.01–0.07	0.03 (0.01); 0–0.07	0.04 (0.01); 0.01–0.08	0.03 (0.01); 0.01–0.08	0.06 (0.02); 0.01–0.11	0.09 (0.03); 0.03–0.19
**GEV F**	–	–	0.04 (0.01); 0.02–0.07	0.04 (0.01); 0.02–0.08	–	–	–	0.04 (0.01); 0.01–0.08
**GEV G**	–	–	0.04 (0.01); 0.02–0.07	0.04 (0.01); 0.02–0.08	–	–	–	0.04 (0.01); 0.01–0.08

Each cell in the table lists the mean, standard deviation, minimum and maximum values (mean (SD); min-max) of the following microstate temporal parameters: Occurrence, Duration, Coverage and Global Explained Variance (GEV) of classes A, B, C, D, E, F, and G for participants from the*Khula*and*Germina*cohorts for visits 1 through 4.

### 3.2 Developmental changes in microstate dynamics

**Fig. 2.**
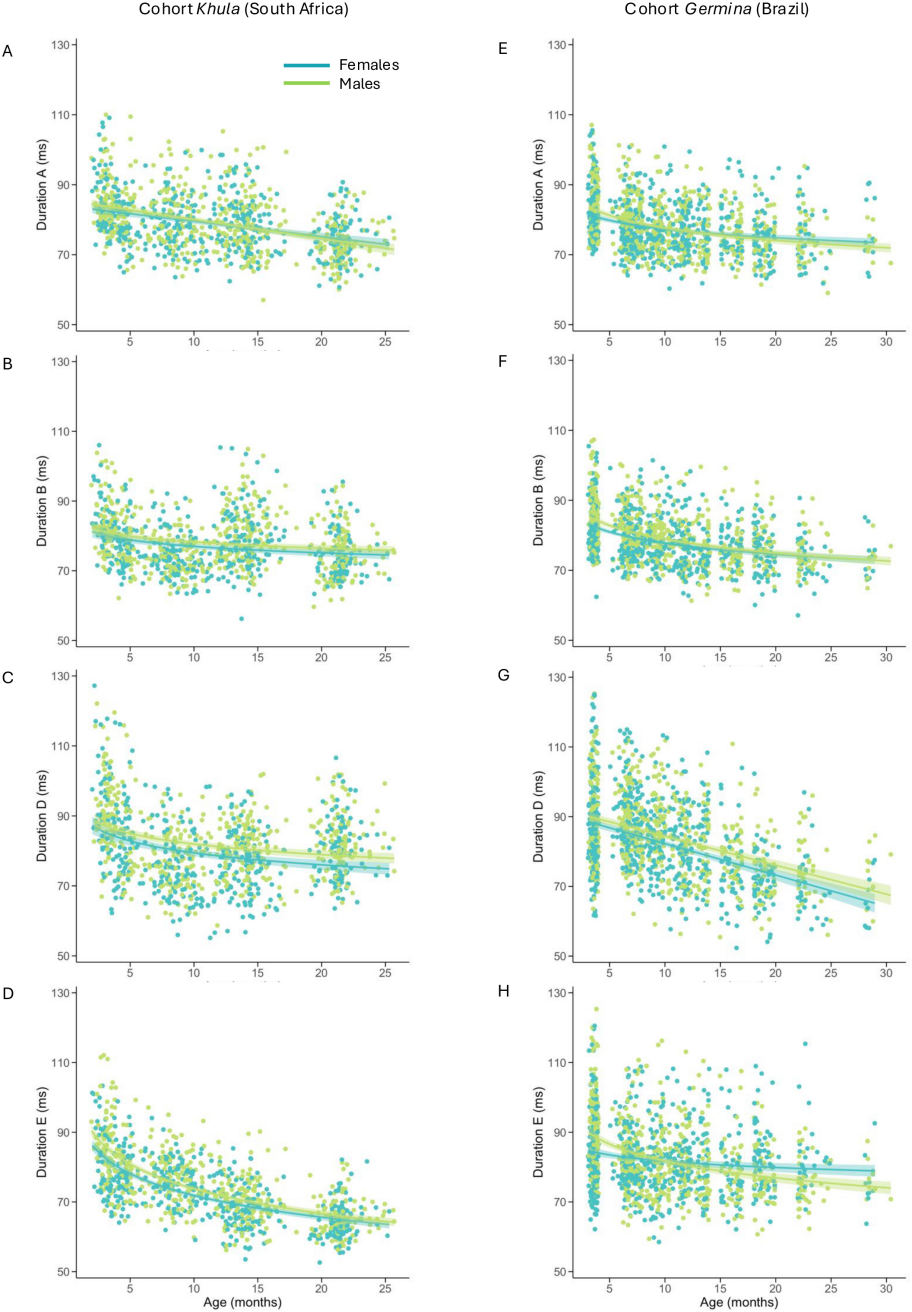
Developmental trajectories of microstate durations through infancy. The figure shows cohort-specific age-associated changes in the duration of microstates A (2A, 2E), B (2B, 2F), D (3C, 3G), and E (3D, 3H), categorized by sex (light green = males; teal = females). Linear or logarithmic regression lines and their 95% confidence intervals (shaded) are shown in each plot.

To assess developmental changes in microstate dynamics over the first 2 years of life, primary analyses focused on changes in microstate occurrences (i.e., average number of times a given microstate was present per second) and durations (i.e., average time in milliseconds that a given microstate was uninterruptedly present within the EEG) over this age range (Figs. 2and3). Specifically, occurrence and duration features were assessed for the microstate classes present across all four time points (i.e., microstates A, B, D, and E) using linear mixed-effects models. Models included number of EEG segments retained as covariates of no interest in addition to effects of sex and age (linear). Additional non-linear age-related changes were tested for via inclusion of added non-linear terms (quadratic or logarithmic) and retained if either non-linear term improved model fit. We observed widespread developmental changes in microstates’ duration and occurrence statistics (Tables 4and5) which are detailed for each microstate below.

**Fig. 3.**
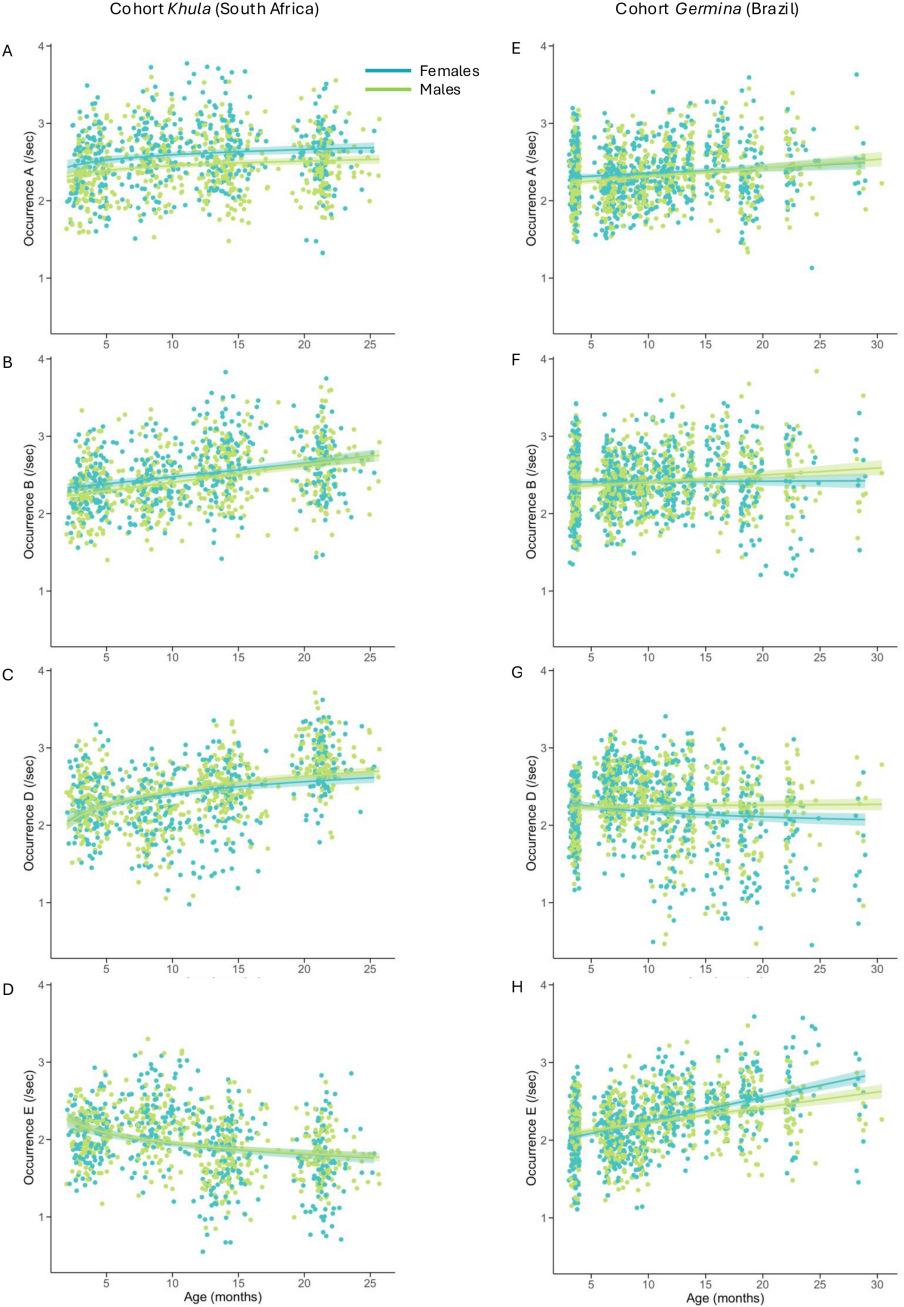
Developmental trajectories of microstate occurrences through infancy. The figure shows cohort-specific age-associated changes in the occurrence of microstates A (3A, 3E), B (3B, 3F), D (3C, 3G), and E (3D, 3H), categorized by sex (light green = males; teal = females). Linear or logarithmic regression lines and their 95% confidence intervals (shaded) are shown in each plot.

**Table 4.** Developmental changes in microstate durations.

Cohort	Microstate parameter	Model (age)	Predictor	Estimate ( *b* )	Std. Error	*p* -value	Effect size (Cohen’s *f* ^ 2^ )
***Khula***	durationA	linear	age	-0.505	0.036	**<0.001**	** 0.25
			sex	0.305	0.582	0.600	<0.01
			segments	-0.042	0.038	0.268	<0.01
	durationB	linear + log	age	0.364	0.131	**0.006**	<0.01
			log (age)	-5.925	1.283	**<0.001**	* 0.03
			sex	1.172	0.504	**0.021**	* 0.02
			segments	0.012	0.011	0.265	<0.01
	durationD	linear + log	age	1.902	0.153	**<0.001**	** 0.2
			log (age)	-22.407	1.502	**<0.001**	** 0.29
			sex	2.461	0.745	**0.001**	* 0.04
			segments	0.000	0.013	0.982	<0.01
	durationE	linear + log	age	-0.259	0.113	**0.022**	<0.01
			log (age)	-7.160	1.108	**<0.001**	* 0.05
			sex	1.944	0.540	**<0.001**	* 0.04
			segments	0.027	0.010	**0.006**	<0.01
***Germina***	durationA	linear + log	age	0.234	0.106	**0.027**	<0.01
			log (age)	-6.575	1.031	**<0.001**	* 0.04
			sex	0.654	0.440	0.137	<0.01
			segments	-0.029	0.017	0.083	<0.01
	durationB	linear + log	age	0.094	0.101	0.350	<0.01
			log (age)	-5.958	0.981	**<0.001**	* 0.04
			sex	1.274	0.400	**0.002**	* 0.02
			segments	0.032	0.016	**0.043**	<0.01
	durationD	linear	age	-0.880	0.047	**<0.001**	** 0.31
			sex	2.193	0.631	**0.005**	*0.02
			segments	-0.034	0.025	0.180	<0.01
	durationE	linear + log	age	0.310	0.147	**0.035**	<0.01
			log (age)	-7.507	1.430	**<0.001**	* 0.03
			sex	0.970	0.621	0.119	<0.01
			segments	-0.018	0.024	0.454	<0.01

The statistics for fixed effects of the model outputs (durationA/B/D/E) have been listed as estimates and their standard errors for microstate durations being predicted by age and sex of infants, and the number of segments retained from each EEG recording. The effect sizes for the significant regressions (*p*< 0.05, highlighted in bold) were estimated using Cohen’s*f*^ 2^categorized as small (denoted by *) if*f *^2^≥ 0.02, medium (denoted by **) if*f*^ 2^≥ 0.15, or large (denoted by ***) if*f *^2^≥ 0.35 byCohen (1988). SeeSupplementary Table S13for marginal R^2^values.

**Table 5.** Developmental changes in microstate occurrences.

Cohort	Microstate parameter	Model (age)	Predictor	Estimate ( *b* )	Std. Error	*p* -value	Effect size (Cohen’s *f* ^ 2^ )
***Khula***	occurrenceA	linear + log	age	-0.033	0.007	**<0.001**	* 0.03
			log (age)	0.394	0.066	**<0.001**	* 0.04
			sex	-0.138	0.027	**<0.001**	* 0.09
			segments	-0.001	0.001	**0.046**	<0.01
	occurrenceB	linear	age	0.020	0.002	**<0.001**	** 0.16
			sex	-0.063	0.026	**0.016**	*0.02
			segments	-0.001	0.001	0.084	<0.01
	occurrenceD	linear + log	age	0.088	0.006	**<0.001**	** 0.26
			log (age)	-0.579	0.061	**<0.001**	* 0.11
			sex	0.036	0.027	0.184	<0.01
			segments	-0.001	0.001	**0.036**	<0.01
	occurrenceE	linear + log	age	-0.050	0.007	**<0.001**	* 0.07
			log (age)	0.270	0.066	**<0.001**	* 0.02
			sex	0.016	0.028	0.581	<0.01
			segments	0.000	0.001	0.896	<0.01
***Germina***	occurrenceA	linear	age	0.009	0.002	**<0.001**	* 0.03
			sex	-0.041	0.020	**0.041**	<0.01
			segments	-0.002	0.001	**0.018**	<0.01
	occurrenceB	linear	age	0.005	0.002	**0.004**	<0.01
			sex	-0.009	0.023	0.680	<0.01
			segments	0.001	0.001	0.160	<0.01
	occurrenceD	linear + log	age	-0.076	0.007	**<0.001**	* 0.12
			log (age)	0.661	0.064	**<0.001**	* 0.1
			sex	0.052	0.028	0.060	<0.01
			segments	-0.001	0.001	0.496	<0.01
	occurrenceE	linear	age	0.026	0.002	**<0.001**	** 0.26
			sex	-0.019	0.021	0.353	<0.01
			segments	0.001	0.001	0.364	<0.01

The statistics for fixed effects of the model outputs (occurrenceA/B/D/E) have been listed as estimates and their standard errors for microstate occurrences being predicted by age and sex of infants, and the number of segments retained from each EEG recording. The effect sizes for the significant regressions (*p*< 0.05, highlighted in bold) were estimated using Cohen’s*f*^ 2^categorized as small (denoted by *) if*f*^ 2^≥ 0.02, medium (denoted by **) if*f*^ 2^≥ 0.15, or large (denoted by ***) if*f*^ 2^≥ 0.35 byCohen (1988). SeeSupplementary Table S13for marginal R^2^values.

#### 3.2.1 Microstate A

Microstate A (auditory network) durations significantly decreased with age across both cohorts over infancy. The linear mixed effects model in*Khula*(South Africa) showed a moderate-to-strong negative effect of linear age on microstate A duration (*b*_linear_age_= -0.505,*p*< 0.001,*f *^2^= 0.25). The model in*Germina*(Brazil) revealed a non-linear negative effect of age on microstate A duration such that duration significantly decreased with increasing age, with faster rates of change in early infancy. The logarithmic age term was negatively associated with a small-to-moderate effect size (*b*_log_age_= -6.575,*p*< 0.001,*f *^2^= 0.04), while the linear age term had a positive effect on microstate A duration (*b*_linear_age_= 0.234,*p*= 0.027,*f *^2^< 0.01). Infant sex did not differentiate developmental trajectories for microstate A duration in either*Khula*or*Germina*(*p*> 0.05).

Though microstate A decreased in duration, it significantly increased its occurrence with age. This relation was non-linear within individuals in the*Khula*cohort (*b*_log_age_= 0.394,*p*< 0.001,*f *^2^= 0.04; (*b*_linear_age_= -0.033,*p*< 0.001,*f*^ 2^= 0.03), increasing most rapidly over early infancy. In the*Germina*cohort, occurrence significantly increased with linear age (*b*_linear_age_= 0.009,*p*< 0.001,*f*^ 2^= 0.03). Moreover, the infant’s biological sex was a significant predictor of the occurrence of microstate A in both*Khula*(*b*= –0.138,*p*< 0.001,*f ²*= 0.09) and*Germina*(*b*= –0.041,*p*= 0.041,*f ²*< 0.01), with a small-to-moderate effect size in*Khula*and a statistically significant but negligible effect size in*Germina*. The males demonstrated fewer microstate A occurrences than females, engaging this largescale brain network less frequently each second.

#### 3.2.2 Microstate B

Microstate B (visual network) durations decreased non-linearly with age across both cohorts over infancy. With duration decreasing most steeply over the first year, there was a statistically significant negative association with the logarithmic age term (*b*_log_age_= -5.925,*p*< 0.001,*f *^2^= 0.03) and a negligibly significant positive effect of the linear age term (*b*_linear_age_= 0.364,*p*= 0.006,*f*^ 2^< 0.01). In*Germina,*we observed a statistically significant decreasing duration of microstate B with logarithmic age (*b*_log_age_= -5.958,*p*< 0.001,*f*^ 2^= 0.04). Additionally, infant sex had a significant effect in predicting the duration of microstate B in both*Khula*(*b*= 1.172,*p*= 0.021,*f*^ 2^= 0.02) and*Germina*(*b*= 1.274,*p*= 0.002,*f *^2^= 0.02), such that males had significantly longer microstate durations than females, indicating slower transitions from microstate B to other microstate configurations in males.

Microstate B demonstrated a significant linear increase in occurrence over the first 2 years in both cohorts, with a moderate effect size in*Khula*(*b*_linear_age_= 0.020,*p*< 0.001,*f *^2^= 0.16) and a statistically significant but negligible effect size in*Germina*(*b*_linear_age_= 0.005,*p*= 0.004,*f *^2^< 0.01). The occurrence of microstate B was also significantly predicted by infant sex in*Khula*(*b*= -0.063,*p*= 0.016,*f*^2^= 0.02), with males demonstrating fewer occurrences than females.

#### 3.2.3 Microstate D

Microstate D (dorsal attention network) durations significantly decreased with age with large developmental effects in both cohorts. The linear mixed effects model for*Khula*revealed a strong non-linear effect of age on microstate D duration within individuals, with duration showing the most rapid decrease in early infancy (*b*_log_age_= -22.407,*p*< 0.001,*f *^2^= 0.29;*b*_linear_age_= 1.902,*p*< 0.001,*f *^2^= 0.2). In*Germina*, a strong negative effect of linear age was observed on microstate D duration within individuals (*b*_linear_age_= -0.880,*p*< 0.001,*f*^ 2^= 0.31). Additionally, infant sex had a significant effect in predicting the duration of microstate D in both*Khula*(*b*= 2.461,*p*= 0.001,*f*^ 2^= 0.04) and*Germina*(*b*= 2.193,*p*= 0.005,*f*^ 2^= 0.02), indicating significantly longer microstate durations in males than in females.

Microstate D occurrence demonstrated different non-linear developmental patterns across cohorts. In*Khula*, there was a significant non-linear increase in microstate D occurrence associated with age, showing a slower increase in the second year (*b*_log_age_= -0.579,*p*< 0.001,*f *^2^= 0.11;*b*_linear_age_= 0.088,*p*< 0.001,*f*^2^= 0.26). However, the model for*Germina*suggested an early increase of microstate D occurrence followed by a decrease over the second year. We observed a positive effect of logarithmic age (*b*_log_age_= 0.661,*p*< 0.001,*f*^ 2^= 0.10) but a negative association with the linear age (*b*_linear_age_= -0.076,*p*< 0.001,*f*^ 2^= 0.12). Infant sex did not differentiate developmental trajectories for microstate D occurrence in either*Khula*or*Germina*(*p*> 0.05).

#### 3.2.4 Microstate E

Microstate E (salience network) showed statistically significant non-linearly decreasing duration with age in*Khula*, with the fastest rate of change in early infancy (*b*_log_age_= -7.160,*p*< 0.001,*f *^2^= 0.05;*b*_linear_age_= -0.259,*p*= 0.022,*f*^ 2^< 0.01). Statistically significant non-linearly decreasing duration of microstate E was also seen in*Germina*(*b*_log_age_= -7.507,*p*< 0.001,*f *^2^= 0.03;*b*_linear_age_= 0.310,*p*= 0.035,*f*^ 2^< 0.01). Moreover, infant sex in*Khula*significantly predicted the duration of microstate E (*b*= 1.944,*p*< 0.001,*f*^ 2^= 0.04), where males had longer microstate durations than females.

Microstate E occurrence demonstrated different non-linear developmental patterns across cohorts. The mixed effects model in*Khula*indicated a significant non-linear decrease in occurrence (*b*_log_age_= 0.270,*p*< 0.001,*f *^2^= 0.02;*b*_linear_age_= -0.050,*p*< 0.001,*f*^ 2^= 0.07), whereas in*Germina*, an age-associated statistically significant linear increase was observed in the occurrence of microstate E (*b*_linear_age_= 0.026,*p*< 0.001,*f*^ 2^= 0.26). Infant sex did not differentiate developmental trajectories of microstate E occurrence (*p*> 0.05) for both*Khula*and*Germina*.

## 4 Discussion

Our study provides a comprehensive characterization of resting-state EEG microstate dynamics during the first 2 years of life when the most rapid and significant development of largescale functional networks takes place. We have investigated the number and type of microstates across this developmental window in two large geographically, culturally, and socioeconomically diverse cohorts from Cape Town, South Africa (*Khula*, N = 318), and São Paulo, Brazil (*Germina*, N = 536). We found consistent microstate classes emerged between the two disparate cohorts over development. For microstate classes that were present throughout the first 2 years, we also evaluated developmental changes in their durations and occurrences to understand largescale network dynamics in this period. We found both consistent and site-unique developmental patterns across microstate dynamics, suggesting these indices of largescale networks reflect both foundational and context-sensitive neurodevelopmental dynamics. These results inform our understanding about a key period of brain development in the following ways.

First, we found highly conserved patterns of microstate classes across two geographically and culturally diverse cohorts using data-driven clustering. Specifically, our approach for obtaining microstate maps suggested the presence of microstates A, B, C, D, and E in the first year of life with the emergence of two new microstates F and G (and the absence of microstate C) in the second year of life. Notably, microstate C was identified only in early infancy in both cohorts and may reflect incomplete spatiotemporal segregation and integration of networks in this period. Relatedly, in adult microstate literature where the microstate labeling has been restricted to only the four canonical maps A–D, other microstates are merged and labeled microstate C, especially microstate F because of the spatial correlation as C and F share regions from the Default Mode Network, and microstate E because of shared topographical characteristics despite functional differences (Bréchet et al., 2019;Khanna et al., 2015;Pascual-Marqui et al., 2014;Santarnecchi et al., 2017;Tarailis et al., 2021,^2023^;Zanesco, 2024). Indeed, microstate C re-emerged if we forced a four-class microstate solution within these developmental data, except for the fourth*Khula*time point which produced an unclassifiable map (seeSupplementary Tables S3 and S4). Here the consistent developmental shift from microstates C to F and the instability of microstate C in a data-driven classification context suggests incomplete integration of the default mode network in infancy. However, the overall pattern of microstate classifications suggests consistent largescale network configurations, and developmental shifts in these configurations are observable in early life across disparate contexts.

Next, we found conserved evidence that faster transitions between largescale brain topographies emerge over infancy, especially over the first year of life. That is, the duration of all microstates (A, B, D, and E) significantly decreased within individuals over the first 2 years of life in both cohorts. Moreover, the best-fitting models for these developmental changes overwhelmingly included nonlinear terms (all except microstate A in*Khula*and microstate D in*Germina*), indicating the steepest decreases in the durations of microstates occurred over early infancy across both cohorts. These reductions in durations, reflecting faster transitions between largescale brain topographies, suggest more efficient sub-second level interactions between underlying largescale network configurations over infancy (Khanna et al., 2015;Michel & Koenig, 2018). Such developmental trajectories are consistent with previously reported fMRI findings in sleep indicating that infants undergo rapid increases in functional segregation and integration across brain regions, contributing to more efficient neural processing in largescale networks on the scale of seconds to minutes (Doria et al., 2010;Gao, Alcauter, Smith, et al., 2015;Gao et al., 2009;Lin et al., 2008). Moreover, the white matter tracts involved in sensory and cognitive networks undergo substantial maturation and myelination over the first 2 years of life to support faster conduction speed of neural signals and more efficient neural communication across distant brain regions (Deoni et al., 2016;Dubois et al., 2014;Gao et al., 2015;Lebel & Deoni, 2018;Schilling et al., 2023). These significant structural changes potentially facilitate the significantly shorter microstate durations that we observed in our results. Future research should examine how these early structural and functional indices of largescale networks co-develop.

Furthermore, sensory microstates showed consistent patterns of development between cohorts. Specifically, microstates A and B, associated with the auditory and visual network, respectively, became briefer with age, but recurred more frequently within each second. This temporal pattern aligns with existing research indicating that sensory networks emerge early postnatally and are among the first to mature during infancy (Doria et al., 2010;Fair et al., 2009;Gao et al., 2015;Michel & Koenig, 2018;Smyser et al., 2011). Here we show that this functional maturation in awake behaving infants includes rapid, frequent instances of these sensory network configurations each second, which may aid in the detection of relevant sensory information from infants’ environments. How these networks’ frequent, brief configurations at the sub-second level support learning and higher-order neurodevelopment should be explored further (Britz et al., 2010;Hill et al., 2023).

In contrast, the attention and task switching-related microstates, microstates D (dorsal attention network) and E (salience network), exhibited divergent developmental trajectories between the*Khula*and*Germina*cohorts. The dorsal attention network is crucial for voluntary attentional control, while the salience network is involved in detecting and integrating stimuli that are emotionally or behaviorally relevant (Menon & Uddin, 2010;Seeley et al., 2007;Zhou et al., 2018). Microstate D increased in occurrence with age in the*Khula*cohort but decreased in the*Germina*cohort, while microstate E displayed the opposite pattern. The contrasting trajectories may reflect environmental or socio-cultural factors that shape higher-order brain network engagement in different ways across contexts. For example, prior research has suggested that differences in early caregiving environments, linguistic exposure, and socio-economic conditions can influence the pace of brain network maturation and integration (Bagdasarov et al., 2022;Holz et al., 2023;Khazaei et al., 2021;Tarailis et al., 2021). This pattern of findings highlights the need for more culturally and geographically diverse studies investigating neurodevelopment to unravel how geocultural conditions shape brain network organization and dynamics in early life.

In addition to these developmental changes, our results revealed small yet significant sex-related differences in several microstates’ dynamics. Compared with females, males across both cohorts showed significantly longer durations of microstate B, microstate D, and microstate E (in*Khula*only). We also observed significantly fewer occurrences of microstates A (*Khula*and*Germina*) and B (*Khula*only) in males as compared with females. Overall, males exhibited slightly longer durations and fewer occurrences of largely sensory microstates as compared with females, indicating slower temporal transitions between brain network states over infancy. This set of findings is consistent with prior studies indicating sex differences in neural maturation rates, particularly in brain regions associated with attention and sensory processing where males can lag females developmentally (Bagdasarov et al., 2022;Tomescu et al., 2018). Together, these different dynamics suggest potential sex differences in how largescale networks supporting sensory processing are engaged functionally at the sub-second level during early neurodevelopment. Such differences may have implications for understanding sex-specific developmental trajectories in cognitive domains, as slower transitions between neural states have been linked to later development of cognitive flexibility and executive function (Das et al., 2022;Hill et al., 2023). Longitudinal studies beyond age 2 years may shed light on how these foundational sex-dependent largescale network differences scaffold higher-order neurocognitive development.

While our study provides important insights into the developmental trajectories of EEG microstates across the first 2 years of life, several limitations and avenues for future research warrant consideration. While the use of fixed-duration epochs (2 seconds) ensured consistency in microstate analysis, the total number of segments per recording was inherently limited by the relatively short EEG acquisition time (2–3 minutes), which is a common constraint in developmental research to accommodate young participants. Although the number of retained segments significantly influenced the occurrence of microstates A (in both*Khula*and*Germina*) and D (*Khula*), as well as the duration of microstates B (*Germina*) and E (*Khula*), it is important to note that all of these effects showed negligible effect sizes (*f*² < 0.01). This suggests that while statistically detectable, these particular microstate parameter variations may hold limited biological or clinical significance. Second, our investigation is limited to the age range of 2 to 30 months. Extending studies beyond this range longitudinally is crucial to capture the continued development of largescale brain networks and their relationship with emerging cognitive and behavioral skills in early childhood. Third, while we examined microstate dynamics across two geoculturally distinct cohorts, future studies should include broader sociocultural contexts and cohorts with greater within-cohort variability in SES measures to further assess the generalizability and potential socioeconomic influences on developmental patterns of microstates. Although the microstate research community has some consensus on the association between microstate classes and the specific largescale functional networks, individual microstates cannot be entirely reduced to a single, well-defined function (Milz et al., 2017;Zappasodi et al., 2019). Instead, their interpretation should consider factors like the participants’ subjective states, the specific task or questionnaires administered (Tarailis et al., 2023). Future research could integrate EEG with other neuroimaging modalities like fMRI, to uncover the spatial correlates of microstate dynamics and to provide a more comprehensive understanding of early brain network organization (Nishida et al., 2013;Rajkumar et al., 2021). Moreover, our study focused on resting-state EEG, which limits knowledge about network dynamics in active cognition. Exploring task-related microstate dynamics in awake, behaving infants presents an exciting opportunity to elucidate how these networks support emerging cognitive and behavioral skills during development. Such integrative and multimodal approaches will help to advance our understanding of the temporal, spatial, and functional aspects of largescale brain network maturation in early life.

Our results illustrate both the early emergence and significant developmental changes in largescale brain networks’ functional dynamics at the sub-second level across infancy. By examining microstate properties across longitudinal cohorts diverse in geography, culture, and socioeconomic factors, our study underscores the robustness of EEG microstates as markers of early foundational brain development. Microstate trajectories across cohorts suggest some foundational sensory network functional development proceeds similarly across these contextual differences, while networks serving higher-order cognitive processes like attention are sensitive to context. Together these longitudinal results provide new insights into how largescale functional brain network dynamics and development unfold in early life.

## Supplementary Material

Supplementary Material

## Data Availability

Access to the datasets used in this study requires a formal data-sharing agreement subject to institutional and ethical guidelines. The custom script used for generation of microstates and computation of all microstate parameters is available athttps://github.com/PINE-Lab/HAPPE/blob/master/3.%20generate/generateMicroStates.m.
