# Supplementary material for: Spatiotemporal dynamics of EEG microstate networks over the first two years of life: A multi-cohort longitudinal study
Source: Imaging Neurosci (Camb). 2025 Jun 27;3:IMAG.a.59. doi: 10.1162/IMAG.a.59 (PMC12319982; doi:10.1162/IMAG.a.59)
Supplement: Supplementary Material [file imag.a.59_supp.pdf]

Supplementary Materials

Supplementary Table S1. HAPPE v4 Preprocessing Script Parameters

Preprocessing Parameter Type	***Parameter Used***
**Density**	High (>30 channels)
**Resting State or Task**	Resting state
**Acquisition Layout**	128 channel EGI HydroCel Geodesic Sensor Net
**Channels**	All except E1, E8, E14, E17, E21, E25, E32, E38, E43, E44, E48, E49, E56, E68, E73, E81, E88, E94, E99, E107, E113, E114, E119, E120, E121, E125, E126, E127, E128
**Line Noise Frequency**	50 Hz (South Africa) / 60 Hz (Brazil)
**Line Noise Reduction Method**	CleanLine - Default
**Resample**	Off
**Filter - Lowpass Cutoff**	40 Hz
**Filter - Highpass Cutoff**	1 Hz
**Filter Type**	EEGLAB's FIR
**Bad Channel Detection**	On
**Bad Channel Detection Method**	Default
**Wavelet Thresholding**	Default
**Wavelet Threshold Rule**	Hard
**MuscIL**	On
**Segmentation**	On
**Segment Length**	2 seconds
**Interpolation**	Off
**Segment Rejection**	On
**Segment Rejection Method**	Amplitude criteria only
**Minimum Segment Rejection Threshold**	-150 μV
**Maximum Segment Rejection Threshold**	150 μV
**Re-Reference Method**	Average

Supplementary Table S2. EEG Quality Control Measures

Cohort		*Khula*				*Germina*			
EEG quality control measure		Visit 1 (N=242)	Visit 2 (N=249)	Visit 3 (N=261)	Visit 4 (N=218)	Visit 1 (N=349)	Visit 2 (N=444)	Visit 3 (N=350)	Visit 4 (N=201)
% channels selected		89.72 (6.9); 57.58 - 100	93.75 (4.84); 71.72 - 100	92.63 (5.3); 64.65 - 100	91.83 (6.3); 64.65 - 100	85.43 (6.9); 41 - 97	89.58 (5.21); 61 - 98	90.22 (4.25); 76 - 99	90.8 (4.89); 68 - 99
% trials retained		94.34 (15.46); 16.48 - 100	94.86 (15.2); 16.48 - 100	95.87 (10.71); 26.37 - 100	96.33 (12.5); 23.08 - 100	93.81 (15.34); 23.08 - 100	90.06 (18.33); 24.19 - 100	89.35 (19.3); 24.59 - 100	93.44 (15.63); 28.33 - 100
Correlation of data pre- vs post- wavelet thresholding (Pearson's r)	at 2 Hz	0.41 (0.2); 0.00005 - 0.87	0.34 (0.21); 0.001 - 0.84	0.38 (0.22); 0.003 - 0.9	0.48 (0.22); 0.01 - 0.95	0.33 (0.22); 0.01 - 0.9	0.35 (0.24); 0.0001 - 0.91	0.32 (0.25); 0.003 - 0.92	0.37 (0.24); 0.003 - 0.96
	at 5 Hz	0.63 (0.23); 0.0002 - 0.99	0.61 (0.27); 0.004 - 0.95	0.64 (0.25); 0.02 - 0.97	0.76 (0.21); 0.02 - 0.99	0.52 (0.26); 0.01 - 0.96	0.49 (0.27); 0.0009 - 0.95	0.48 (0.29); 0.01 - 0.97	0.54 (0.28); 0.01 - 0.98
	at 8 Hz	0.6 (0.24); 0.0003 - 0.98	0.64 (0.26); 0.003 - 0.98	0.7 (0.26); 0.03 - 0.98	0.83 (0.18); 0.02 – 0.99	0.46 (0.26); 0.02 - 0.91	0.49 (0.28); 0.0008 - 0.97	0.53 (0.3); 0.01 - 0.99	0.62 (0.29); 0.01 - 0.99
	at 12 Hz	0.63 (0.24); 0.0007 - 0.98	0.59 (0.27); 0.01 - 0.95	0.63 (0.27); 0.01 - 0.97	0.77 (0.21); 0.01 - 0.99	0.5 (0.26); 0.01 - 0.94	0.5 (0.27); 0.0007 - 0.96	0.48 (0.29); 0.01 - 0.97	0.55 (0.27); 0.01 - 0.98
	at 20 Hz	0.71 (0.21); 0.0002 - 0.99	0.65 (0.25); 0.01 - 0.96	0.67 (0.25); 0.01 - 0.97	0.78 (0.19); 0.01 - 0.99	0.6 (0.24); 0.04 - 0.95	0.59 (0.26); 0.0009 - 0.96	0.56 (0.27); 0.01 - 0.98	0.61 (0.26); 0.02 - 0.98
	at 30 Hz	0.76 (0.2); 0.0001 - 0.99	0.7 (0.24); 0.01 - 0.97	0.71 (0.25); 0.01 - 0.98	0.79 (0.17); 0.03 - 0.99	0.67 (0.23); 0.04 - 0.97	0.67 (0.25); 0.0009 - 0.98	0.62 (0.26); 0.01 - 0.99	0.67 (0.25); 0.03 - 0.98

Supplementary Figure S3. 4-class microstate solutions

Cohort	Age (months)	No. of Subjects	Microstate A	Microstate B	Microstate C	Microstate D	Microstate E	Unclassified Microstate
**Khula (South Africa)**	2-6	242	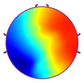	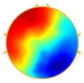	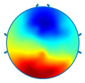	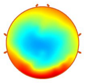		
	5-12	249	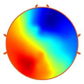	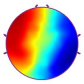	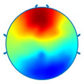	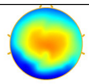		
	12-18	261	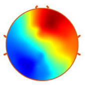	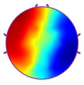	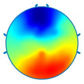	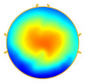		
	19-26	218	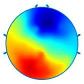	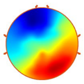		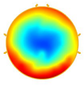		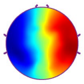
**Germina (Brazil)**	3-4	349	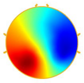	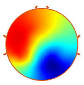	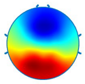	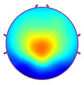		
	5-10	444	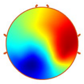	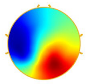	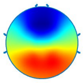	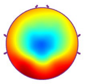		
	10-17	350	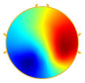	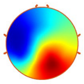	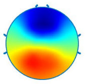	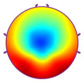		
	18-30	201	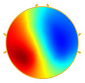	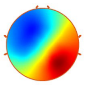	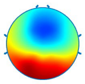		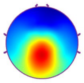	

Supplementary Table S4. Summary statistics of microstate (*k=4*) parameters

Cohort	**Khula (South Africa)**				**Germina (Brazil)**			
Timepoint	**T1 (2-6mo)**	**T2 (5-12mo)**	**T3 (12-18mo)**	**T4 (19-26mo)**	**T1 (3-4mo)**	**T2 (5-10mo)**	**T3 (10-17mo)**	**T4 (18-30mo)**
occurrence_A	2.54.(0.35); 1.76-3.68	2.97.(0.37); 2.00-4.06	3.05.(0.35); 2.21-3.97	3.37.(0.34); 2.19-4.13	2.58.(0.33); 1.58-3.50	2.66.(0.29); 1.78-3.77	2.77.(0.34); 1.43-4.06	2.90.(0.37); 1.60-4.22
occurrence_B	2.58(0.36); 1.70-3.50	2.46(0.33); 1.53-3.37	2.59(0.31); 1.55-3.52	3.28(0.35); 2.34-4.16	2.69(0.33); 1.86-3.60	2.63(0.29); 1.71-3.40	2.90(0.32); 2.05-3.69	2.96(0.41); 1.80-4.14
occurrence_C	2.99 (0.35); 1.80-4.00	3.27(0.35); 2.43-4.25	3.33(0.33); 2.37-4.14		2.93(0.34); 1.80-3.71	3.03(0.35); 1.93-3.98	3.23(0.36); 2.23-4.12	3.28(0.45); 1.69-4.16
occurrence_D	2.59 (0.31); 1.71-3.49	2.76(0.42); 1.24-3.65	2.74(0.51); 0.78-4.06	2.93(0.41); 0.58-3.84	2.45(0.35); 1.05-3.38	2.60(0.41); 0.63-3.61	2.60(0.59); 0.43-3.69	
occurrence_E								2.69(0.59); 0.42-3.92
occurrence(u)				2.59(0.30); 1.73-3.33				
duration_A	85.90(12.38); 66.27-216.39	85.76 (8.98); 62.27-118.00	84.28 (9.33); 65.06-127.80	87.53 (9.14); 68.69-119.40	86.89 (8.44); 72.17-125.01	86.19 (9.64); 67.40-139.33	81.20 (8.84); 57.73-131.14	79.54(9.50); 64.73-144.24
duration_B	86.37 (9.54); 70.26-153.72	75.59 (6.39); 62.72-115.54	75.79 (6.54); 64.00-106.95	84.82 (9.12); 66.32-116.43	89.53 (8.35); 68.97-122.90	85.76 (8.30); 70.84-120.34	84.13 (9.40); 63.30-128.58	79.84 (7.64); 63.54-119.16
duration_C	102.06(9.36); 75.23-137.12	96.45(10.11); 73.47-125.01	94.05 (9.77); 68.13-126.84		102.66(10.14); 73.60-137.29	99.62(10.04); 73.76-147.88	94.24(10.13); 73.50-140.42	92.26(12.84); 68.47-167.99
duration_D	97.01(15.47); 67.56-164.35	87.03(11.76); 58.04-134.49	83.73(10.13); 52.99-113.84	80.98(10.11); 58.27-137.60	95.01(16.23); 62.92-170.20	93.06(12.41); 55.05-145.11	85.92(11.85); 52.57-127.50	
duration_E								83.16(14.62); 53.20-171.39
duration(u)				72.06 (5.23); 62.38-93.50				
coverage_A	0.22(0.04); 0.12-0.42	0.26 (0.05); 0.14-0.43	0.26 (0.05); 0.16-0.44	0.30 (0.05); 0.17-0.46	0.22 (0.03); 0.14-0.36	0.23 (0.04); 0.14-0.41	0.23 (0.04); 0.09-0.39	0.23 (0.05); 0.12-0.45
coverage_B	0.22(0.04); 0.14-0.40	0.19 (0.03); 0.11-0.31	0.20 (0.03); 0.11-0.29	0.28 (0.05); 0.17-0.43	0.24 (0.03); 0.14-0.35	0.23 (0.03); 0.16-0.38	0.24 (0.04); 0.14-0.37	0.24 (0.04); 0.13-0.38
coverage_C	0.31(0.05); 0.16-0.45	0.32(0.05); 0.18-0.48	0.31(0.05); 0.16-0.51		0.30(0.04); 0.17-0.43	0.30(0.05); 0.18-0.54	0.30(0.05); 0.17-0.47	0.30(0.06); 0.13-0.47
coverage_D	0.25(0.06); 0.14-0.42	0.24(0.06); 0.07-0.38	0.23(0.06); 0.04-0.39	0.24(0.05); 0.03-0.39	0.23(0.06); 0.07-0.44	0.24(0.05); 0.05-0.42	0.23(0.07); 0.03-0.38	
coverage_E								0.23(0.07); 0.02-0.58
coverage(u)				0.19(0.03); 0.13-0.27				
GEV_A	0.06(0.02); 0.02-0.16	0.10(0.04); 0.04-0.27	0.10(0.04); 0.04-0.23	0.15(0.05); 0.05-0.34	0.05(0.02); 0.02-0.12	0.06(0.02); 0.02-0.19	0.06(0.02); 0.02-0.15	0.07(0.02); 0.02-0.20
GEV_B	0.06(0.03); 0.02-0.16	0.05(0.02); 0.02-0.11	0.06(0.02); 0.02-0.12	0.13(0.04); 0.05-0.31	0.06(0.02); 0.02-0.16	0.06(0.02); 0.02-0.12	0.07(0.02); 0.03-0.19	0.07(0.03); 0.02-0.18
GEV_C	0.13(0.05); 0.03-0.26	0.15(0.05); 0.06-0.30	0.16(0.05); 0.05-0.42		0.11(0.04); 0.03-0.25	0.11(0.04); 0.04-0.40	0.12(0.04); 0.04-0.26	0.13(0.05); 0.02-0.41
GEV_D	0.06(0.02); 0.02-0.16	0.07(0.02); 0.01-0.17	0.07(0.03); 0.01-0.17	0.08(0.02); 0.00-0.16	0.05(0.02); 0.01-0.12	0.06(0.02); 0.01-0.14	0.06(0.03); 0.00-0.27	
GEV_E								0.07(0.03); 0.00-0.27
GEV(u)				0.05(0.02); 0.02-0.12				

Supplementary Figure S5. Measures of fit plot for Khula timepoint 1 (age 2-6 months)

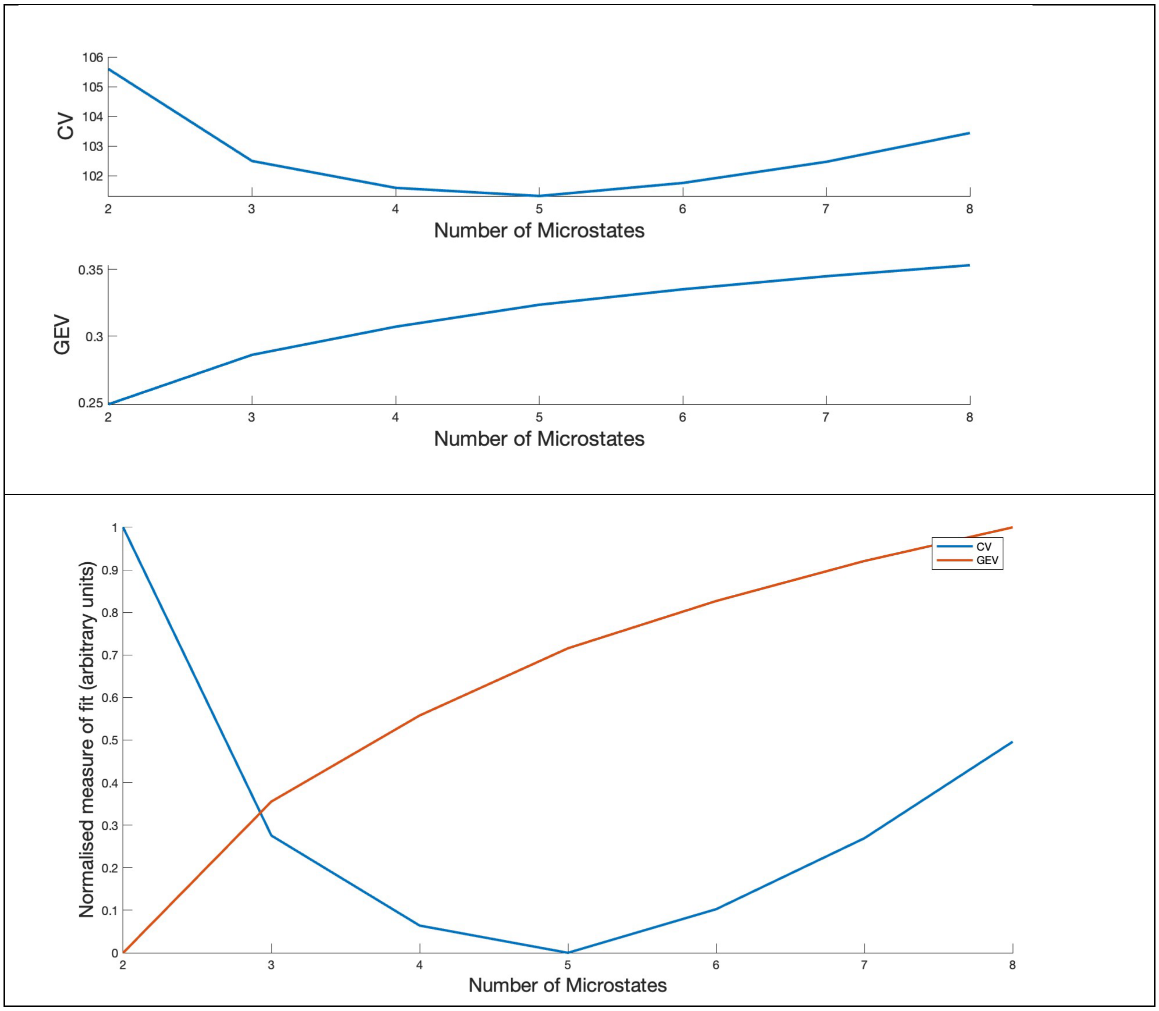

Supplementary Figure S6. Measures of fit plot for Khula timepoint 2 (age 5-12 months)

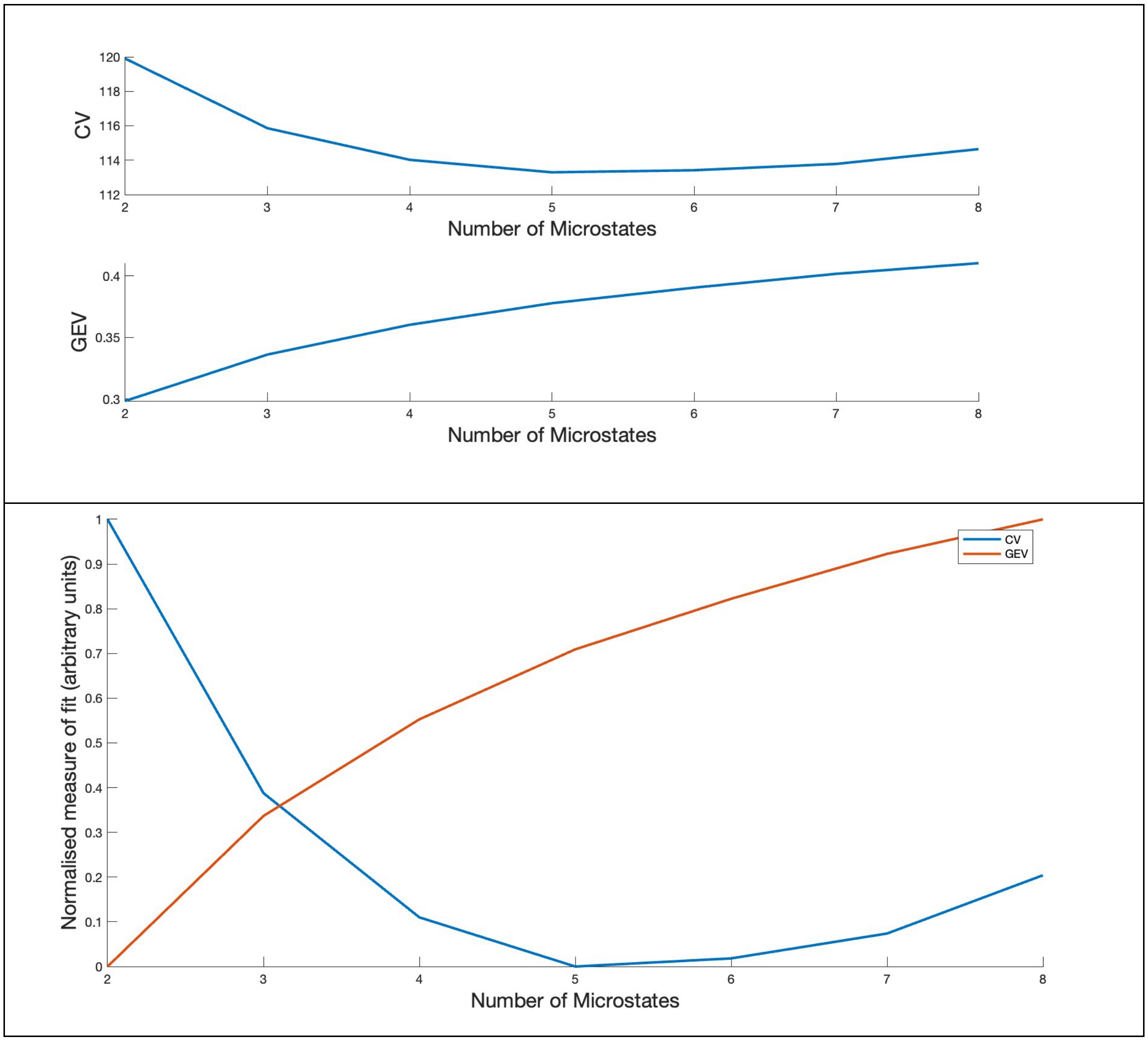

Supplementary Figure S7. Measures of fit plot for Khula timepoint 3 (age 12-18 months)

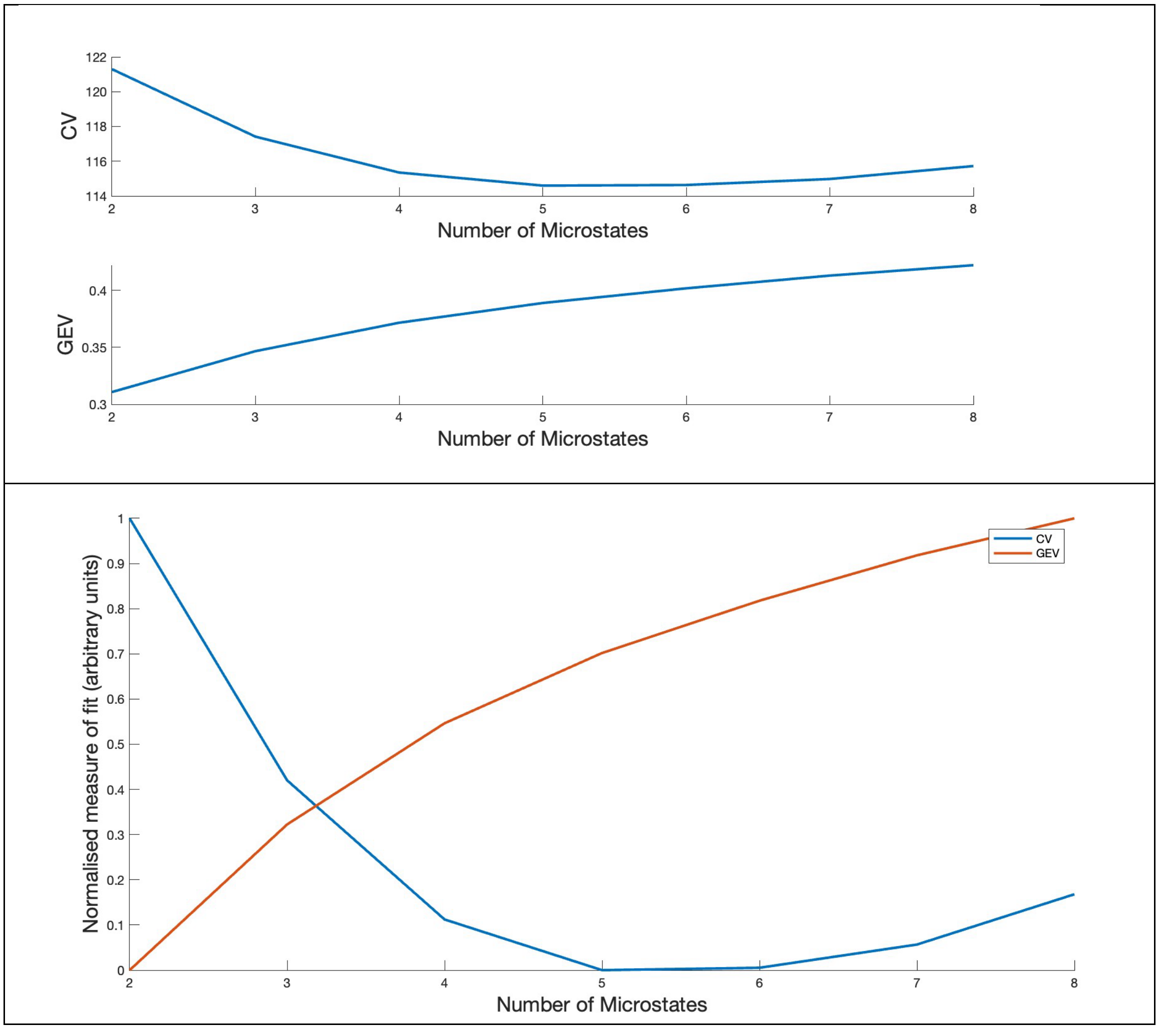

Supplementary Figure S8. Measures of fit plot for Khula timepoint 4 (age 19-26 months)

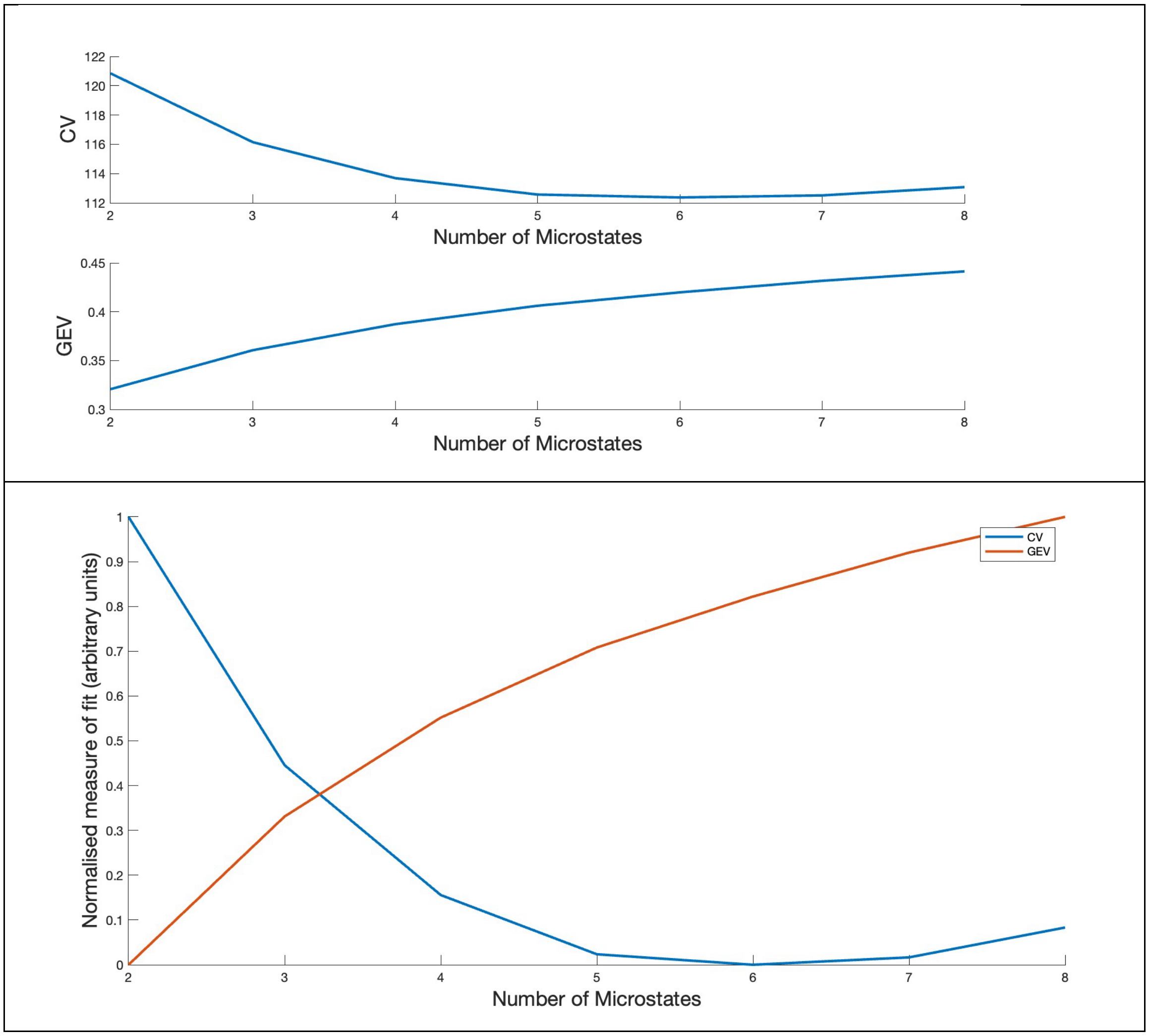

Supplementary Figure S9. Measures of fit plot for Germina timepoint 1 (age 3-4 months)

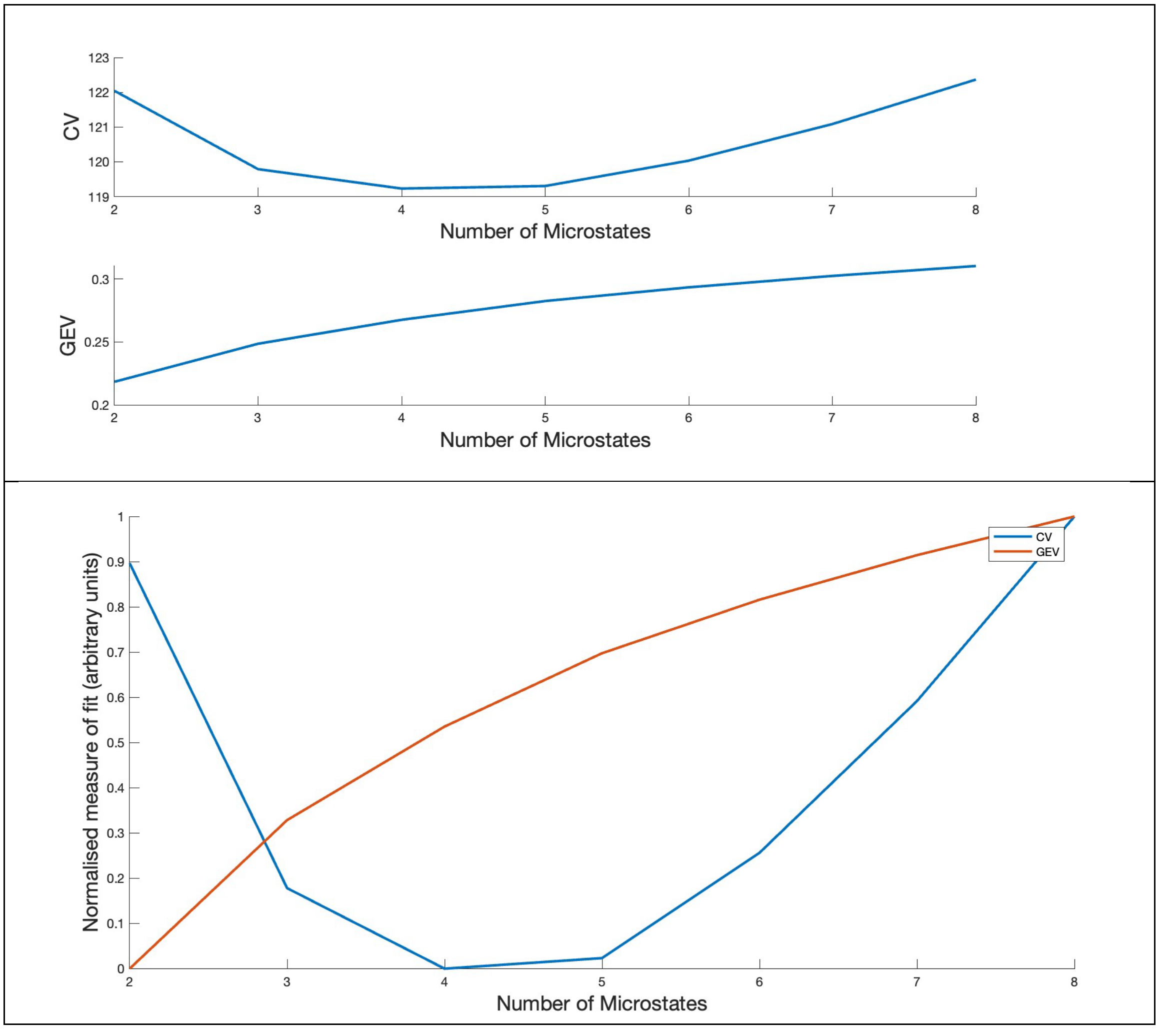

Supplementary Figure S10. Measures of fit plot for Germina timepoint 2 (age 5-10 months)

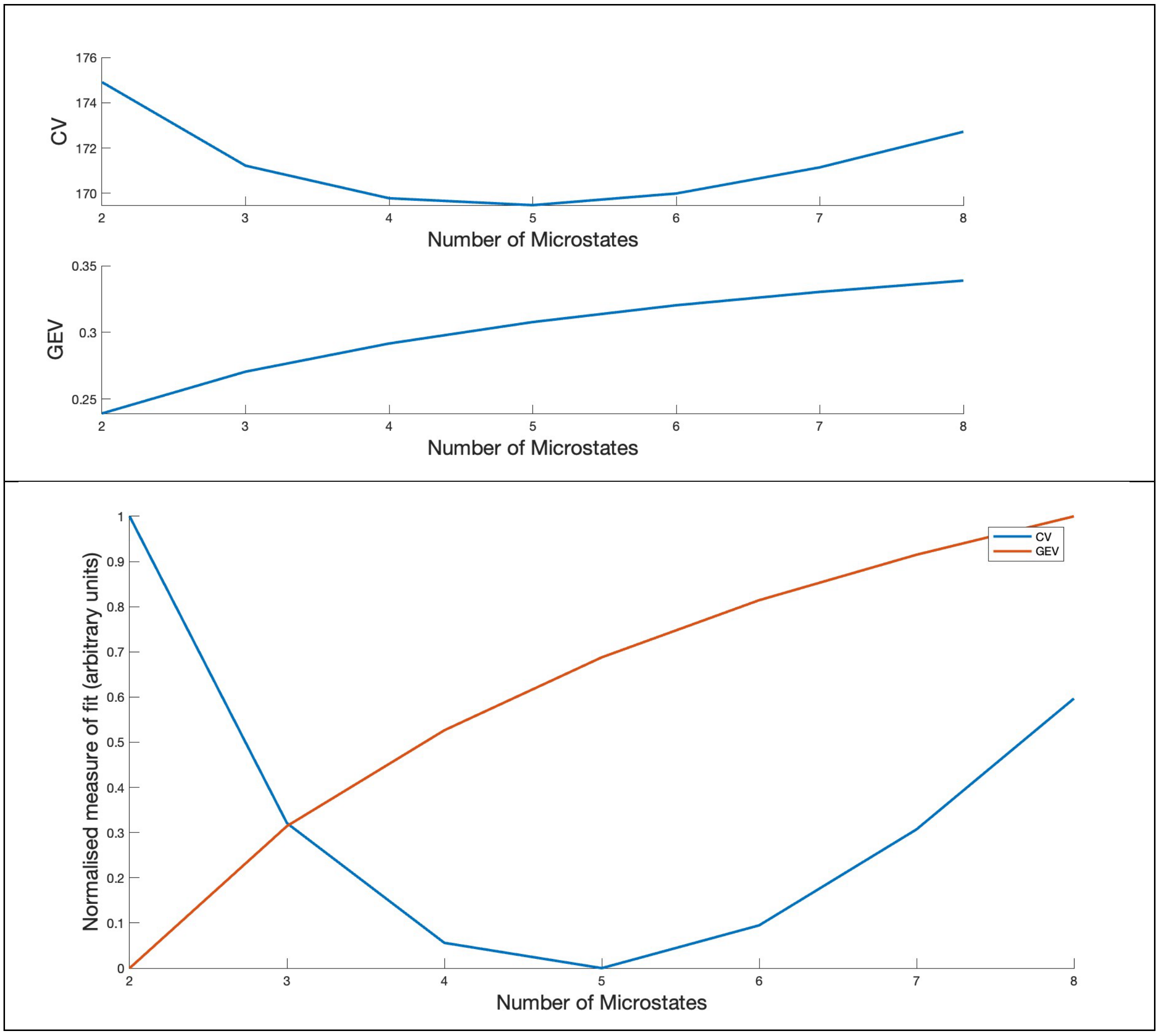

Supplementary Figure S11. Measures of fit plot for Germina timepoint 3 (age 10-17 months)

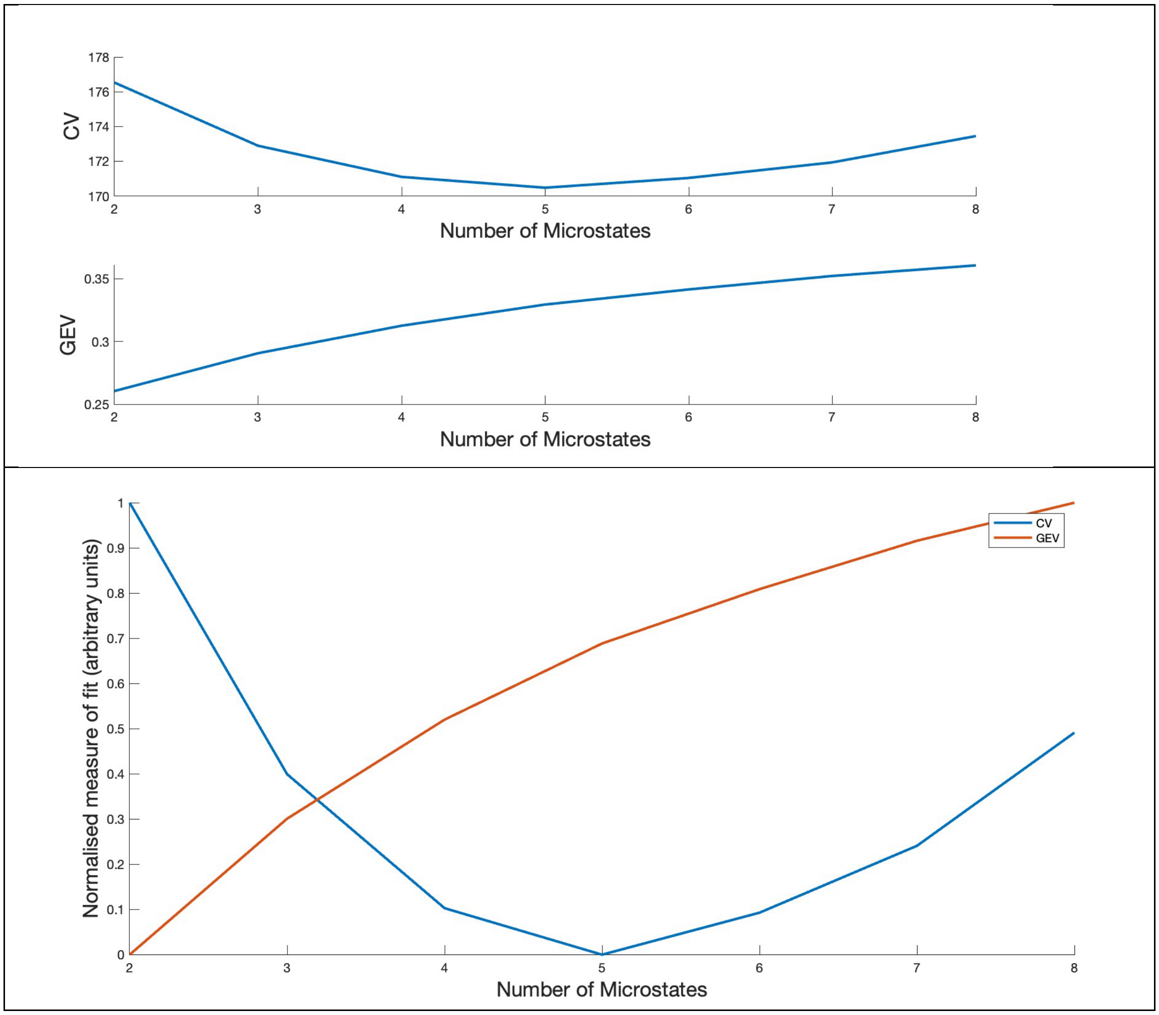

Supplementary Figure S12. Measures of fit plot for Germina timepoint 4 (age 18-30 months)

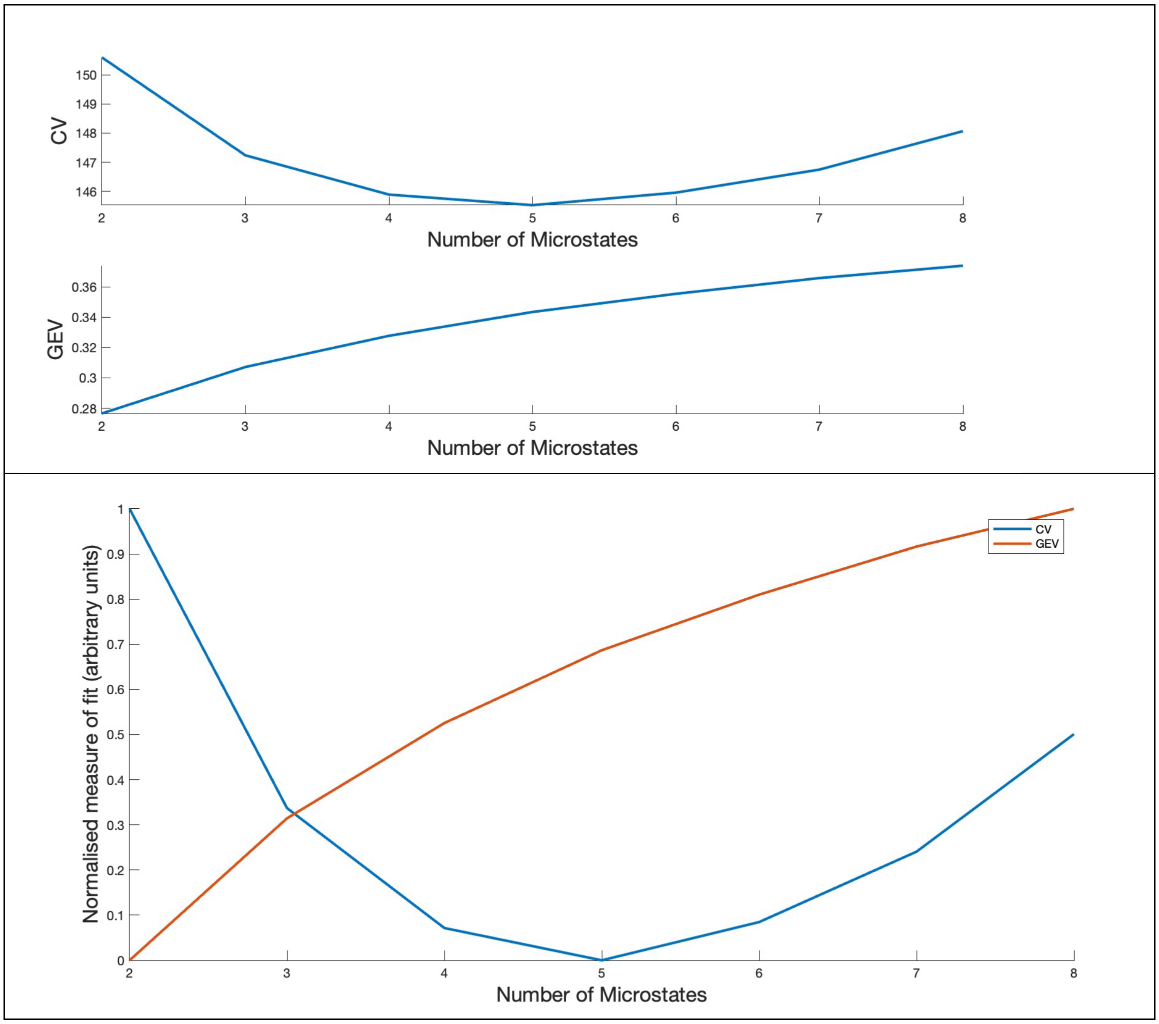

Supplementary Table S13. Marginal R^2 values for effect sizes of all fixed effects in the lmer model predicting microstate durations and occurrences

Microstate parameter	**Khula**	**Germina**
**occurrence_A**	0.079	0.031
**occurrence_B**	0.121	0.007
**occurrence_D**	0.286	0.087
**occurrence_E**	0.141	0.176
**duration_A**	0.154	0.130
**duration_B**	0.063	0.192
**duration_D**	0.205	0.212
**duration_E**	0.469	0.080
